# Conformational Characterization of the Co-Activator Binding Site Revealed the Mechanism to Achieve the Bioactive State of FXR

**DOI:** 10.3389/fmolb.2021.658312

**Published:** 2021-08-31

**Authors:** Anita Kumari, Lovika Mittal, Mitul Srivastava, Dharam Pal Pathak, Shailendra Asthana

**Affiliations:** ^1^ Translational Health Science and Technology Institute (THSTI), Faridabad, India; ^2^ Department of Pharmaceutical Chemistry, Delhi Pharmaceutical Sciences and Research University (DPSRU), New Delhi, India; ^3^ Delhi Institute of Pharmaceutical Sciences and Research (DIPSAR), New Delhi, India

**Keywords:** farnesoid X receptor, agonist, molecular dynamics simulation, binding free energy calculations, principal component analysis

## Abstract

FXR bioactive states are responsible for the regulation of metabolic pathways, which are modulated by agonists and co-activators. The synergy between agonist binding and ‘co-activator’ recruitment is highly conformationally driven. The characterization of conformational dynamics is essential for mechanistic and therapeutic understanding. To shed light on the conformational ensembles, dynamics, and structural determinants that govern the activation process of FXR, molecular dynamic (MD) simulation is employed. Atomic insights into the ligand binding domain (LBD) of FXR revealed significant differences in inter/intra molecular bonding patterns, leading to structural anomalies in different systems of FXR. The sole presence of an agonist or ‘co-activator’ fails to achieve the essential bioactive conformation of FXR. However, the presence of both establishes the bioactive conformation of FXR as they modulate the internal wiring of key residues that coordinate allosteric structural transitions and their activity. We provide a precise description of critical residue positioning during conformational changes that elucidate the synergy between its binding partners to achieve an FXR activation state. Our study offers insights into the associated modulation occurring in FXR at bound and unbound forms. Thereafter, we also identified hot-spots that are critical to arrest the activation mechanism of FXR that would be helpful for the rational design of its agonists.

## Introduction

**FIGURE 1:**
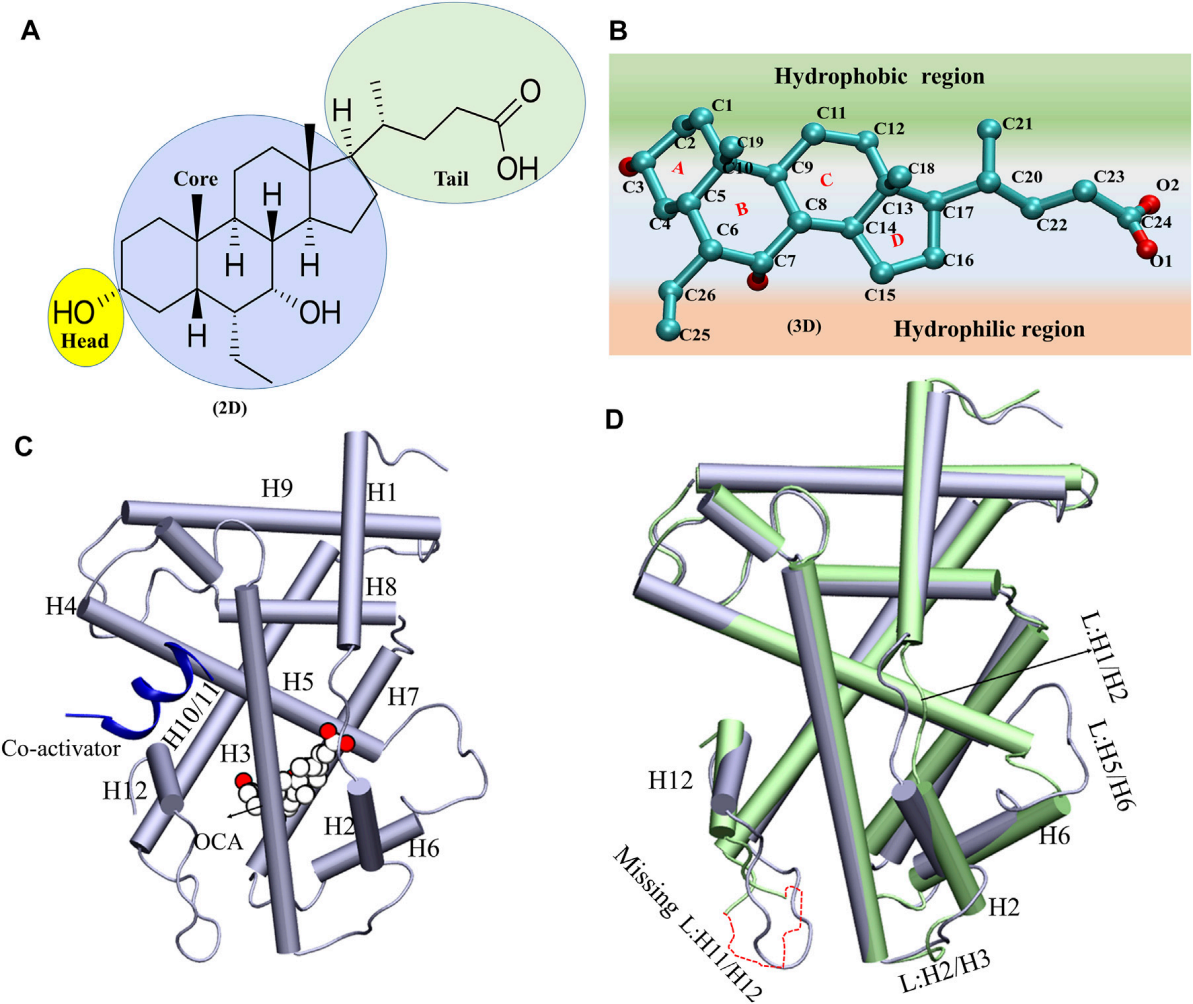
The schematic presentation of the OCA and overlay of the system A and Systems D. **(A,B)** The 2D and 3D representations of ‘OCA’. The different regions of ‘OCA’ were highlighted in different colors. **(C)** The structure of ligand binding domain of FXR. The binding of ‘OCA’ (VDW: white) and ‘co-activator’ (cartoon: blue) are highlighted. **(D)** The superimposition of crystal structures of System C (PDB: ID 5Q0K: in lime) and System D (PDB: ID 1 OSV: ice blue) and induce changes in the structure are mentioned.

Upon bile acid (BA) binding, FXR regulates a network of genes in synthesis, uptake, and secretion along with intestinal absorption, thus regulating the level of BAs in the cells. An abnormal BA metabolism is associated with liver injury, metabolic disorders, cardiovascular and cardiovascular and digestive system diseases (Li and Chiang, 2014; Chiang, 2017). FXR is a nuclear receptor that belongs to the NR superfamily and is predominantly found in the liver, intestine, and kidney (Makishima et al., 1999; Parks et al., 1999; Wang et al., 1999; Aranda and Pascual, 2001). FXR is essential in regulating the network of genes involved in maintaining BA and lipid homeostasis (Sinal et al., 2000; Kumari et al., 2020) and, therefore, has a considerable pharmacological relevance (Zhang and Edwards, 2008; Hollman et al., 2012; Arab et al., 2017). Significant work has been carried out to discover many synthetic molecules viz. steroidal and non-steroidal agonists for the FXR. Accordingly, the *first-in-class* FXR agonist 6α-ethyl-CDCA (2, 6-ECDCA, INT-747, obeticholic acid, OCA) has gained approval for primary biliary cirrhosis (PBC) and is undergoing development for several other liver-related disorders such as NASH and NAFLD (Mudaliar et al., 2013; Neuschwander-Tetri et al., 2015)**.** It is reported that the chemical manipulation on CDCA (chenodeoxycholic acid) scaffold helps to improve potency, efficacy, and metabolic stability of bile acid ligands (Di Leva et al., 2013; Sepe et al., 2015; Festa et al., 2017). Among them, the introduction of an ethyl group at C6 in CDCA makes the 6-EDCA (‘OCA’) (Figures 1A,B) approximately 100-fold more potent than CDCA (Pellicciari et al., 2002). ‘OCA’ is the “*first in class*” selective agonist for FXR having anti cholestatic and hepatoprotective properties (Abenavoli et al., 2018; Connolly et al., 2018). In addition to this, hepatic inflammation and intestinal inflammation can be inhibited by ‘OCA’ induced FXR activation. However, these effects could be problematic in a patient population with an elevated risk for cardiovascular diseases (Hirschfield et al., 2015; Neuschwander-Tetri et al., 2015; Bowlus, 2016; Pencek et al., 2016). Recently, it was reported that ‘OCA’ failed to achieve a first therapy against NASH (AuthorAnonymous, 2020), as it was reported that the complete FXR activation inhibits metabolic cholesterol breakdown and limits bile acid production, resulting in increased cholesterol levels in ‘OCA’ clinical studies (Neuschwander-Tetri et al., 2015). Therefore, it seems that complete and/or pronounced agonism possibly not favorable. Hence, it is essential to discern the binding mechanism, dynamics and determinants of FXR at molecular level.

**GRAPHICAL ABSTRACT:**
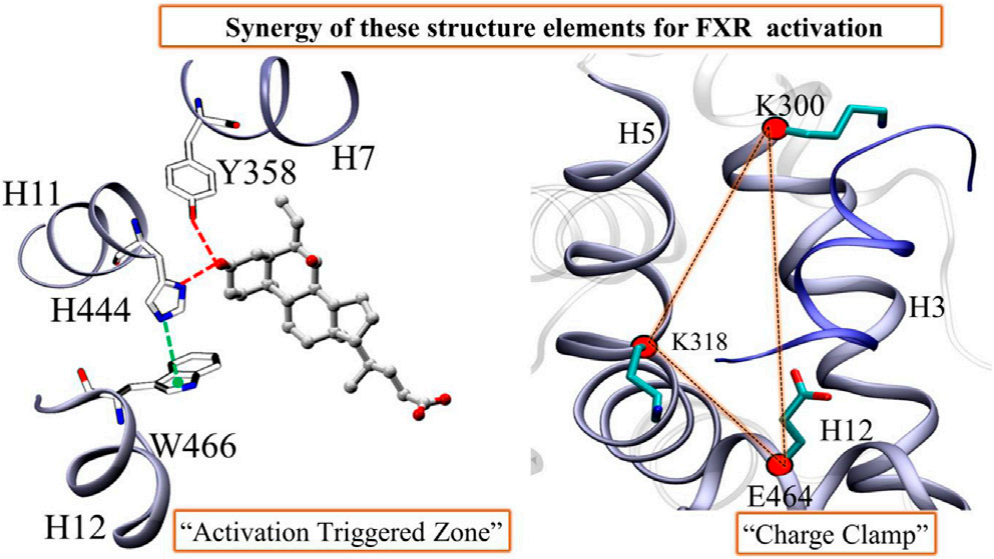
The dynamical synergy between ‘co-activator’ and agonist binding site for FXR activation.

Similarly to other NRs, the FXR protein exhibits a modular structure and contains few autonomous functional domains. It includes an N-terminal region with a ligand-independent activation function (AF1), a highly conserved zinc-finger DNA-binding domain (DBD) that is connected to the LBD by a flexible hinge region (Massafra et al., 2018). Additionally, the LBD contains two well-conserved regions. A signature motif and the AF2 motif are located at the C-terminal end of the LBD, responsible for the ligand-dependent transactivation function. In recent years, a considerable number of crystallographic structures of the LBD of several NRs have appeared in the literature, which suggested that upon agonist binding to FXR, it results in a large conformational rearrangement of FXR, causing the dissociation of co-repressors and the recruitment of ‘co-activator’ which promote the transcriptional initiation (Downes et al., 2003; Costantino et al., 2005; Merk et al., 2019). The crystal comparison of apo- and agonist-bound structures help to identify the key residues and structural determinants for FXR agonism. The static picture from the X-ray structures indicates that significant conformational changes were observed to establish a connection between the apo form and the active state of FXR (bounded with agonist and ‘co-activator’). The co-crystal structure of FXR with ‘OCA’ (PDB-ID: 1OSV) has revealed that helix H12 adopts the https://www.sciencedirect.com/topics/biochemistry-genetics-and-molecular-biology/agonist agonistic conformation and stabilizes the ‘co-activator’ peptide binding (Mi et al., 2003). The binding of ‘OCA’ recruits the helix H12 against the helices 3, 4, and 10, corresponding to the “active state” of FXR, where the helix H12 stabilizes the binding of the ‘co-activator’ (Figure 1C). It seems that the ‘OCA’ has a higher affinity between BAs due to the placement of the 6α-ethyl group into a hydrophobic cavity between the side chains of I359, F363 and Y366 (Pellicciari et al., 2002). This analysis indicates that the binding of the ‘co-activator’ significantly contributes to the stabilization of the FXR+OCA complex and thereby affects the conformation. It has been observed that the recruitment of that agonist and ‘co-activator’ binding are necessary to produce these significant conformational changes and induces a loss or gain of interaction networks stabilized through hydrogen bonding and vdW interactions in FXR. Also, the architecture of ‘co-activator’ site and its dynamical synergy with agonist site is not explored in details. Therefore, we are exploring the dynamical changes of FXR with ‘co-activator’ in the presence and absence of agonists (i.e., ‘OCA’) through molecular dynamics simulations. The precise description of the positioning of critical residues during conformational changes will help to elucidate the synergy with its binding partners and how FXR is able to achieve its activation state using MD simulations, MM-GBSA free energy calculations, essential dynamics, and thermodynamic analysis.

## Materials and Method

### Structure Retrieval

The X-ray structures of the FXR complexes with ‘OCA’ only with a ‘co-activator’ and without any binder (APO) (Supplementary Table S1) were retrieved from Protein Data Bank (Bank, 2021). The crystal structure of human FXR (PDB-ID: 5Q0K) bound with a ‘co-activator’, and rat FXR (PDB-ID: 1OSV) which is bound with ‘OCA’ and ‘co-activator’ both is used for comparative analysis. Since the binding of the coactivator SRC1 (KDHQLLRYLLDKD) in human FXR is similar the binding of ‘co-activator’ GRIP-1 (ENALLRYLLDKD) in rat FXR and both ‘co-activators’ share the high homology between them (Wang et al., 1998; Soisson et al., 2008). These peptides shared the conserved LXXLL motif in the sequence. There are also crystal structures available for human FXR with GRIP-1 (Kudlinzki et al., 2019; Merk et al., 2019). Therefore, we have considered the ‘co-activator’ of rat FXR with human FXR for the study to maintain uniformity. Thus, the FXR without ‘OCA’ and ‘co-activator’ is System A and the FXR with ‘co-activator’ is System C. The FXR with ‘OCA’ is System B and FXR with ‘OCA’ and ‘co-activator’ is System D. The ‘OCA’ without protein is System E. The details of all FXR systems are given in Supplementary Table S1. The LBD of FXR consists of 230 amino acids in structure (total length). The residues involved in the interaction are conserved from the comparative analysis of the binding pocket in both human and rat FXR, which were well studied earlier (Downes et al., 2003; Mi et al., 2003; Kemper, 2011).

### Protein Structure Preparation

Here, we have explored four systems, APO-protein of FXR (System A), APO+agonist (System B), APO + ‘co-activator’ (System C), and APO+Agonist + ‘co-activator’ (System D), to identify the transition dynamics between the different conformational states of FXR with its binding partners. Before MD simulation, each targeted protein structure was prepared using the Protein Preparation Wizard encoded in the Schrodinger 3.5 suite (Sastry et al., 2013; Anang et al., 2018; Sarkar et al., 2021). The crystal waters were also removed, and hydrogens were added. The breaks in the crystal structures were interpolated by using the Prime (Sastry et al., 2013) module of the Schrodinger Suite. The capping was done to the uncapped -N and -C termini of the FXR protein. The hydrogen bond optimization was performed using PROPKA (Rostkowski et al., 2011) at pH7, and the restrained minimizations were also done for the systems using OPLS3 (Optimized Potentials for Liquid Simulations) force field (Harder et al., 2016). To study the sequence similarity between the human FXR and rat FXR crystal structures, the multiple sequence alignment (MSA) was performed by using the PRIME module of Maestro (Proteins, 2004; Mittal et al., 2020).

### MD Simulations

All the systems defined above were subjected to MD simulations. The details of the simulated systems are listed in Supplementary Table S2. In total, we have generated 6.5 µs long MD simulations including the triplicates for each system of FXR**.** The general Amber force field (GAFF) and Amber ff14SB force field were used for ligand, ‘co-activator’, and protein. The antechamber was used to automatically calculate charges and atom types for the ligand (‘OCA’) using GAFF (Wang et al., 2004). The different protein systems for FXR were prepared for simulations using the LEaP program implemented in the Amber package (Pradhan et al., 2018). All the energy minimization and MD simulations are carried out by using the *sander* and *pmemd* modules of AMBER16, respectively (Case et al., 2005). In LEaP, the AMBER ff14SB (Maier et al., 2015) force field was assigned to the protein. Counter ions were added to neutralize the system and the protein system was solvated using a TIP3P water model in an orthorhombic box with a span 10 Å from the periphery of the protein. Each system was neutralized by adding counterion ions. Periodic boundary conditions and particle mesh Ewald methods were employed to treat long-range electrostatic interactions (Darden et al., 1993). Hydrogen bonds were constrained by applying the SHAKE algorithm (Ryckaert et al., 1977). The integration time step for all MD simulations was set at 2 fs. The nonbonded cutoff was 8 Å. The solvated models were first minimized with the module *sander* in constant volume by 2,000 cycles of steepest descent minimization followed by 1,000 cycles of conjugate gradient minimization. The systems were then equilibrated for 500ps at 300 K and 1 atm pressure. For MD simulations, isobaric (NPT) conditions were maintained with the target pressure of 1 bar utilizing the Berendsen barostat. The temperature was regulated using a Langevin thermostat. MD was eventually run for 500 ns, and atomic coordinates were saved every 5ps as snapshots. In addition, the study of MD simulation trajectories was carried out. The simulations have been performed using the GPU version of AMBER16. The last 150ns stable trajectories for all four systems were used for the analysis. To evaluate the stability and dynamics of the FXR systems, triplicate all-atom MD simulations were performed using AMBER 16.

### MD Trajectory Analysis

The root mean square deviations (RMSD) of backbone atoms, root mean square fluctuations of Cα atoms (RMSF), salt bridges, solvent-accessible solvent area (SASA), and radius of gyration (Rg) were calculated for whole trajectories by the Tcl scripts implemented in (visual molecular dynamics) VMD (Humphrey et al., 1996) to assess the overall molecular systems stability and fluctuation in the systems. To explore the systems in terms of compactness, the Rg was calculated. SASA was computed for different systems of FXR bound and unbound with ‘OCA’ and ‘co-activator’. Hydrogen bond (HB) analysis was done using CPPTRAJ of AMBER to search the bonds with in the two selection criteria that is an acceptor-donor distance of 3.5 Å, and acceptor … H-donor … Angle cutoff is 120°. We have calculated the HB for the stable of MD trajectories of the simulation. The CPPTRAJ of Amber16 was used for secondary structure analysis (DSSP), principal component analysis (PCA) analysis, and dynamic cross-correlation matrix (DCCM) plot. The DCCM map and the DSSP plots were generated by using the Cα atoms of all FXR systems throughout the MD simulation (Roe and Cheatham, 2013; Manjula et al., 2019). Following that, we have used the plugin for Pymol (Molecular Graphics System, Version 2.0 Schrödinger, LLC), xPyder, which is an interface that provides the 3D depiction of cross-correlations between residues in dynamics (Pasi et al., 2012). The graphs were plotted using XMGRACE (Turner, 2005). To calculate cation–*π* interaction between the residues W466 and H444, the angle between the atoms of CD1@W466-CE1@H444-CH2@W466 were calculated in all systems of FXR (Khandelia and Kaznessis, 2007).

### Cavity Volume Calculations

As the pocket analysis is useful for the study of structural dynamics of the proteins, therefore we have performed the pocket volume analysis of all FXR systems with the help of the POVME2 (Pocket Volume Measurer) algorithm (Durrant et al., 2014; Srivastava et al., 2018). All water molecules and counterions were stripped from the trajectory. Thereafter, the trajectory was aligned, and the frames were extracted from VMD for all the systems, which is used as initial input for this method. Next, we defined the inclusion and exclusion regions where the inclusion region entirely encompassed all the binding-pocket conformations of the trajectory while the exclusion region is an area that does not associate with the pocket. In our systems, we chose Cα atoms of residues M325 and F365 that lie at the center of a cavity and protrude inwards to it to define the inclusion sphere. The volume of a whole pocket was calculated by simply summing the individual volumes associated with each grid point in the inclusion spheres.

### Free Energy Calculations

**SCHEME 1:**
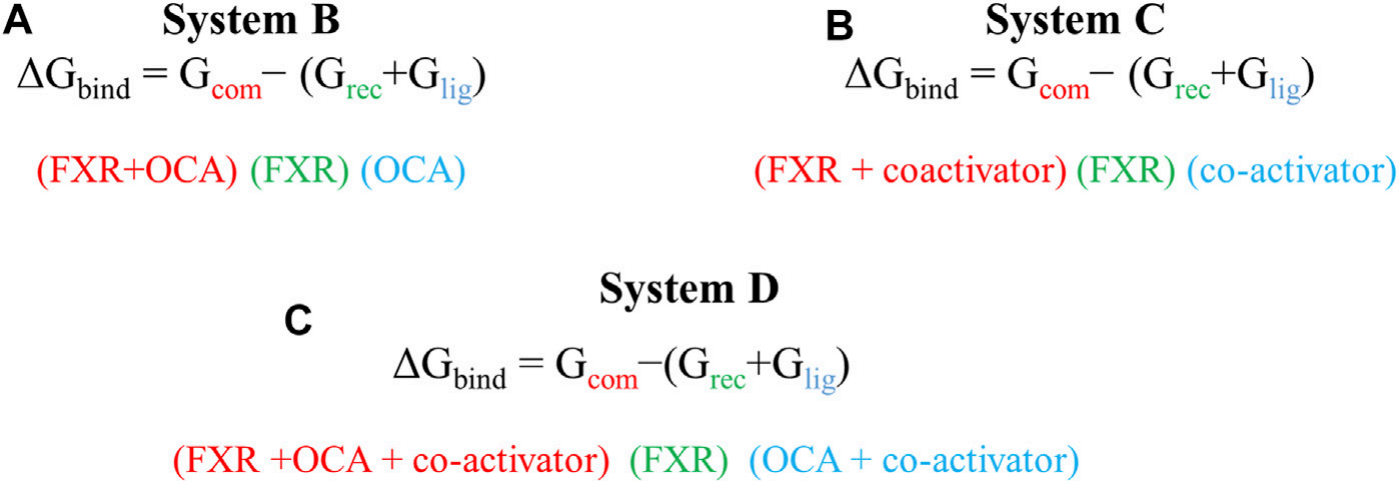
The scheme is used for the calculation of binding energy of ‘OCA’ in presence and absence of ‘co-activator’ in both Systems B and D. The total binding energy of the ‘co-activator’ and ‘OCA’ for System D.

The free energies for FXR systems were calculated by using the MM-GBSA method in AMBER tools and AMBER16 (Chipot and Pohorille, 2007; Suri et al., 2014; Mittal et al., 2019). For this, the frames were extracted from the most stable state from the MD trajectories of all FXR systems. The binding free energy (ΔG_bind_) on each system is evaluated by using the following equation:

$$\Delta {\text{G}}_{\text{bind}}{\;=\text{ G}}_{\text{com}}-\left({\text{G}}_{\text{rec}}{+\text{G}}_{\text{lig}}\right)$$ (1)

where G_com_, G_rec,_ and G_lig_ are the absolute free energies of a complex, receptor, and ligand, respectively, arranged over the equilibrium trajectory. The calculations are performed as per Scheme 1. The free energy, G, for each species can be calculated by using MM-GBSA and MM-PBSA approaches and can be calculated as follows:

$${\text{G}=\text{ E}}_{\text{gas}}{\;+\text{ G}}_{\text{sol}}\;-\text{TS}$$ (2)

$${\text{E}}_{\text{gas}}{\;=\text{ E}}_{\text{int}}{\;+\text{ E}}_{\text{ele}}{\;+\text{ E}}_{\text{vdw}}$$ (3)

$${\text{G}}_{\text{polar}}\text{, PB}\left(\text{GB}\right){\;=\text{ E}}_{\text{ele}}{\;+\text{ G}}_{\text{sol}-\text{polar}}\text{, PB}\left(\text{GB}\right)$$ (4)

$${\text{G}}_{\text{non}-\text{polar}}\text{, PB}\left(\text{GB}\right){\;=\text{ E}}_{\text{vdw}}{\;+\text{ G}}_{\text{sol}-\text{np}}\text{, PB}\left(\text{GB}\right)$$ (5)

$${\text{G}}_{\text{sol}}{\;=\text{ G}}_{\text{PB}\left(\text{GB}\right)}{\;+\text{G}}_{\text{sol}-\text{np}}$$ (6)

$${\text{G}}_{\text{sol}-\text{np}}=\gamma \text{SAS}$$ (7)

Where G is described as a Gibbs free energy, E_gas_ is the gas phase energy which is the sum of internal energy (E_int_), electrostatic interaction (E_ele_), and the van der Waals interaction (E_vdw_). G_sol_ is the solvation free energy is the sum of polar [G_PB(GB)_] and nonpolar contributions (G_sol-np_). It is computed using the parameters defined in the Amber ff14SB force field. G_sol-polar_, PB (GB) is the contribution of polar solvents determined by solving the equations Poisson-Boltzmann (PB) and Generalized-Boltzmann (GB) (Genheden and Ryde, 2015). The overall polar contributions were determined as a summation of the contribution from electrostatics (E_ele_) and polar solvation [G_sol-polar_, PB(GB)]. The sum of the obtained total nonpolar interaction contributions by E_vdw_ and G_sol-np_, PB(GB). G_sol-np_ is the non-polar solvent contribution measured using 0.0072 kcal/mol Å^−2^ (value of constant *γ*) and using a water probe radius of 1.4 Å to determine the solvent-accessible surface area (SASA) (Sitkoff et al., 1994). The dielectric constants were set to 1 and 80, respectively, for solute and solvents. Free energy decomposition in terms of contributions from structural subunits of both binding partners provides insight into the origin of binding on an atomic level.

### Principal Component Analysis

In this work, the PCA, also known as essential dynamics (ED) analysis, is used to study the broad concerted motions in FXR-LBD in their bound and unbound state (Kumari et al., 2021; Mittal et al., 2021; Singh et al., 2021). The analysis was carried out to identify the large-scale average motion of an FXR in all systems by the CPPTRAJ module of AmberTools. The frames were taken from the MD simulation trajectories after the evolution of the systems. To obtain the proper trajectory matrix in PCA, the overall translation or rotation motion was removed by fitting the coordinate data to the average structure. Only the backbone atoms were included during the PCA study. The elements of the positional covariance matrix C are defined by the following equation:

$${\text{C}}_{\text{ij}}\;=\;\langle \left(\text{Xi }-\;\langle \text{Xi}\rangle \right)\left(\text{Xj }-\;\langle \text{Xj}\rangle \right)\rangle \;\;\;\left(\text{i, j}=1\text{,}2\text{,}3\text{……}\text{.,}3\text{N}\right)$$ (8)

where xi and xj are the Cartesian coordinates of the i^th^ and j^th^ Cα atom, N is the number of Cα atoms considered, and ⟨xi⟩ and ⟨xj⟩ represent the time average over all the configurations obtained in the MD simulation (van Aalten et al., 1995; Ivetac and McCammon, 2009). The <> sign indicates the ensemble average of the atomic position in the Cartesian space. Major protein motion that contributes to the overall motion was visualized using the Normal Mode wizard plugin in VMD.

### Free Energy Landscape

The protein global minimum energy can be derived from Free Energy Landscape (FEL). The FEL represents a mapping of all possible conformations which a molecule can adopt during a simulation, together with their corresponding energy typically reported as the Gibbs free energy. The calculation was carried out using the first two principal components (PC1and PC2) obtained from individual trajectories. The first two PCs of the respective systems served as reaction coordinates to generate two-dimensional FEL plots for all FXR systems. This was implemented using the g_sham module of Gromacs (Van Der Spoel et al., 2005).

Gα=-kTlnP(qα)/Pmax(q) (9)

where k is the Boltzman constant, T is the temperature of simulation, P (q α) estimates the probability density function obtained from a histogram of the MD data, and P max(q) is the probability of the most populated state.

### Computational Alanine Scanning

We have carried out CAS for the highlighted residue-wise energy decomposition results to confirm the *hot-spot* amino acids in FXR. The calculations were run on the stable MD trajectory by using the MM-GBSA approach. The amino acid of interest is replaced with alanine, and absolute binding free energy is recalculated. Finally, the difference in the binding free energies of the wild type and mutant, ΔΔG bind, was computed as follows:

$$\Delta \Delta {\text{G}}_{\text{bind}}\;=\;\Delta {\text{G}}_{\text{bind}}\left[\text{Wild Type}\right]\;-\;\Delta {\text{G}}_{\text{bind}}\left[\text{Mutant}\right]$$ (10)

Negative values of ΔΔG bind indicate the favorable contributions of residues in wild type while positive values indicate the unfavorable contributions. The mutant models of all the *hot-spot* residue were generated by using the maestro module.

## Results

As of now, several FXR-LBD crystal structures have been resolved in complex with a range of distinct ligands, which reveals that FXR possesses a highly flexible binding pocket wherein binder dependent conformational changes play an indispensable role to achieve the activation state of FXR (Merk et al., 2019). The reported crystal structures contained only LBD along with agonists/partial-agonists/antagonists and/or co-activators/co-repressors. Since the co-crystal structure represents only a single snapshot of a dynamic binding equilibrium of agonist, ‘co-activator’, or both, it was not sufficient to gain mechanistic understanding.

It remained unclear whether agonist, ‘co-activator’, or both altered the internal wiring of FXR and modulated the structure and function. Further understanding of FXR regulation requires a more in-depth knowledge of the interactions between FXR and its binding partner. It is always interesting to explore the structural determinants responsible to convert active protein to inactive or *vice versa*. The conformational changes that occur during the binding or unbinding processes of different binders induce the essential conformational changes which are required for the transitions of the protein from their different biological states. In total, 13 MD simulations were performed as in triplicates for each system of FXR and we report the outcomes from the consensus of the three simulations (triplicates).

### Exploration of Conformational Changes in the Presence and Absence of ‘OCA’

The structure of ‘OCA’ comprises one 5-membered ring and three six-membered rings fused in Figure 1A. The 2D steroidal rings are named as core region, the OH group as head, and the carboxylic group as a tail region (Figure 1A). The ‘OCA’ displays a convex hydrophobic and a concave hydrophilic face as shown in Figure 1B. Figure1C clearly shows that ring A of the ‘OCA’ faces the C-terminal of helix H12, and this orientation is opposite in other receptors like progesterone, estrogen, testosterone, and glucocorticoids as their ring D faces the helix H12 (Brzozowski et al., 1997; Shiau et al., 1998; Williams and Sigler, 1998; Sack et al., 2001; Bledsoe et al., 2002).

The superimposition of System C and D has shown an average RMSD of backbone atoms of 1.65 Å, indicating the deviations in the backbone atoms of both systems. We have found that helices (H2, H6, and H12) and loops between [H1 and H2 (L: H1/H2), H2 and H3 (L: H2/H3), H5 and H6 (L: H5/H6), and H11 and H12 (L: H11/H12)] have shown deviations in System D relative to the System C. The loop L: H11/H12 is not crystallized in System C, due to low electron density (Figure 1D). It has also been reported that the loop L: H11/H12 is very essential for the stability of the helix H12 position, and the presence of agonists and ‘co-activators’ makes this loop stable in the whole NR family (Costantino et al., 2005). In System D, the helices H2 and H6 were shortened compared to System C, which resulted in significant variations in the loops (L: H2/H3 and L: H5/H6) in the respective systems (Figure 1D). Since these conformational changes in helices and loops reported an impact on binding of ‘OCA’ and ‘co-activator’ therefore, the MD simulations were implemented to discern their mechanism of action (Supplementary Table S1).

### The Dynamical Exploration of FXR in Four Different Systems

**FIGURE 2:**
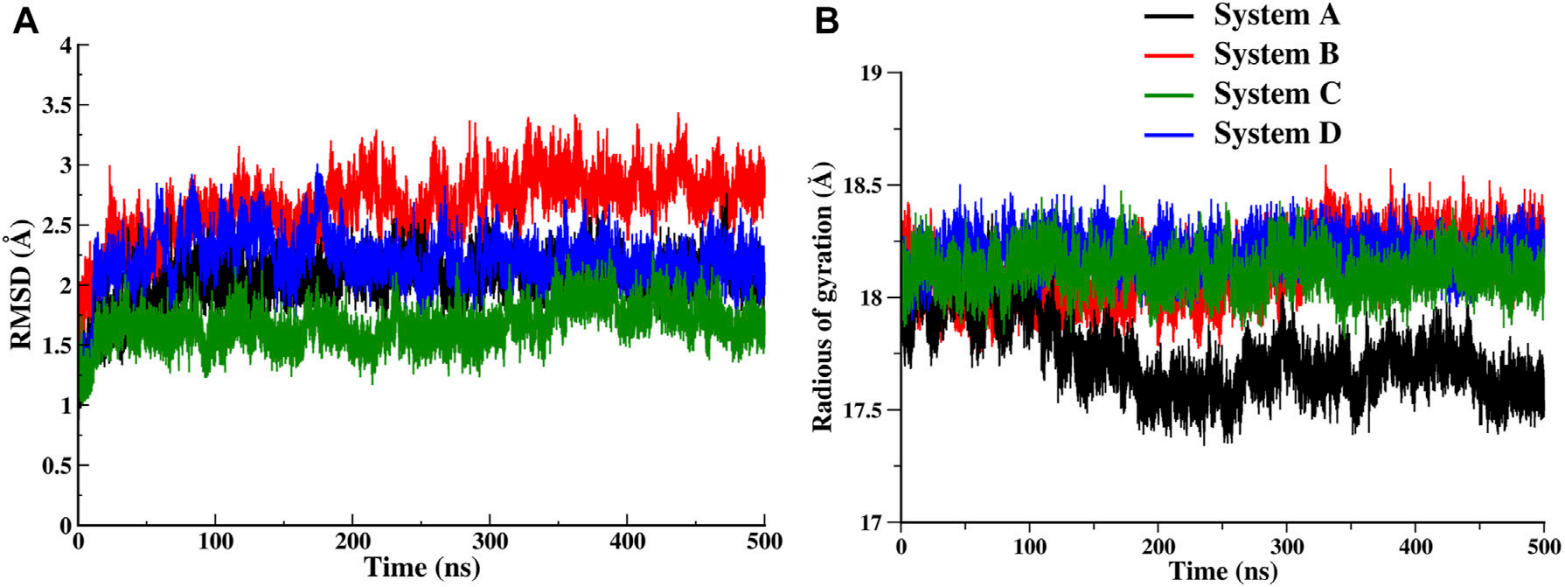
Time series evolution of FXR systems. **(A)** Backbone RMSD during simulation for System A (black), System B (red), System C (green) and System D (blue). **(B)** The Rg plot for all FXR systems.

The time-dependent RMSD of backbone atoms of each simulated system was used to analyze the stability of the systems. As the simulation progressed, each of the systems evolved for a short period of time, from 5 to 40ns, and after that the systems converged, however, the plateau are achieved after 200ns which is consistent till the end of the simulation (Figure 2A). The resulting average values of RMSD are remarkably similar, as expected for each system of three runs, where the backbone atoms of System C appear the most stable (Supplementary Figure S1). The RMSD plots suggest that the binding of ‘OCA’ alone (System B) causes the deviations in the backbone atom from its initial state while the ‘co-activator’ tries to stabilize the FXR protein both as alone and with ‘OCA’ (System C and D). The Rg plot showed that System A became more compact during the simulation as compared to other systems (Figure 2B and Supplementary Table S3). Since System A was formed by removing the ‘co-activator’, its initial Rg was like other systems until ∼100ns but afterward became relatively more compact. This hints that the presence of any binder (‘co-activator’ and/or ligand) causes conformational changes that decrease the compactness of the protein.

**FIGURE 3:**
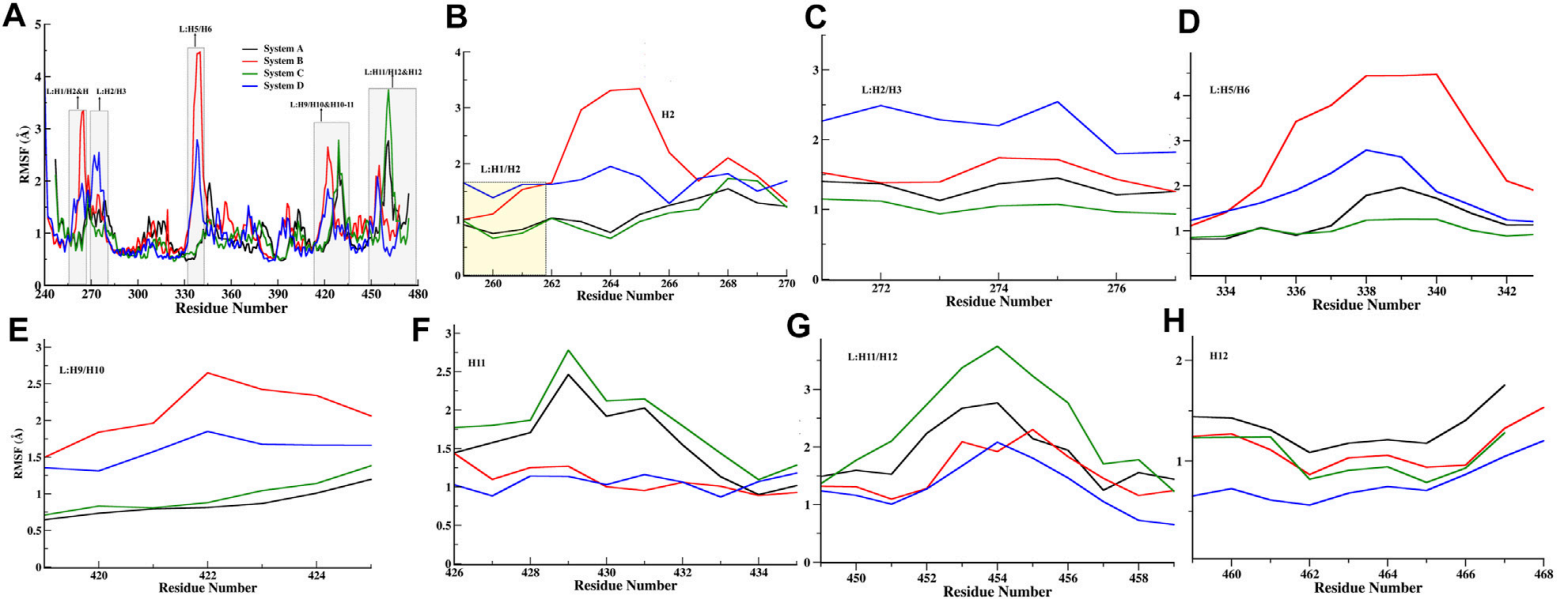
Quantitative analysis of fluctuation from the MD simulation. **(A)** RMSF of Cα atoms of all FXR Systems. The values were presented during the 500ns MD simulations time (ns) scale. The different regions of fluctuations observed in the RMSF plot are mentioned. The region-wise RMSF plots were shown in **(B)** for loop L:H1/H2 and helix H2 region, **(C)** loop between helices 2 and 3 regions (L:H2/H3), **(D)** Loop between helices H5 and H6 region (L:H5/H6), **(E)** loop between H9 and H10 (L:H9/H10), **(F)** helix H11, **(G)** loop between helix 11 and 12 (L:H11/H12) region, and **(H)** helix H12 regions.

Furthermore, the RMSF calculation (of Cα atoms) was performed to identify the regions with high fluctuations, and their average values are summarized in Figure 3A and Supplementary Table S3. The overall RMSF profile reflects that all systems have minimal Cα fluctuations with average values ranging from 0.97 Å to 1.18 Å (Supplementary Table S3). Upon comparing all FXR systems, fluctuations have been observed in helix H2, and loops L: H5/H6 and L: H9/H10 exhibited higher flexibility in Systems B (Figures 3B–E). In System D, the loops L: H1/H2 and L: H2/H3 showed higher fluctuation than the other systems (Figures 3B,C). It seems that ‘OCA’ alone induced fluctuations in helix H2 and in loop areas L: H5/H6, L: H9/H10, whereas the presence of ‘OCA’ with ‘co-activator’ tends to decrease these variations, but the presence of both raises the fluctuation in loops L: H1/H2 and L: H2/H3 (Figures 3B–E). However, the helix H11 and loop L: H11/H12 showed higher fluctuation in System C but these regions experienced the lowest fluctuations in System D (Figures 3F–G). It is also seen that helix H12 has the least fluctuation in System D and highest in System A (Figure 3H). It reflects that binding of ‘OCA’ significantly minimizes the fluctuations in helices H11, H12, and loop L: H11/H12 in the presence of a ‘co-activator’. We also found that the high fluctuation in helix H12 is mainly due to the absence of ‘OCA’ and ‘co-activator’ in System A than other systems (Figure 3H).

Thus, the overall analysis demonstrated that the dynamicity of the FXR highly depends upon the binding of ‘OCA’ and ‘co-activator’ that causes the conformational changes in the FXR. This suggests that the agonist is required to induce a conformational shift in helix H12 so that the ‘co-activator’ can be correctly positioned in the FXR.

### Secondary Structural Changes During the Simulation

To assess secondary structural stability, the secondary structure transitions in each of the FXR systems were analysed during the MD simulation (Supplementary Figure S2). We observe that secondary structure content was retained in all the systems, except the residues located in the helices H2, H6 and loops L: H1/H2, L: H2/H3, L: H5/H6, and L: H11/H12 region of FXR as shown in Supplementary Figures S3–S5. The residues of the loop L: H2/H3 and helix H2 form the stable coil during the simulation in Systems A and C (Supplementary Figure S3). The residues of the loop L: H1/H2 change from the coil to bend secondary structure in System A and B and form the stable secondary structure in Systems C and D. In System B, the residues of the loop L: H2/H3 interchange turn to bend throughout the simulation. Whereas, in System D,these residues form the bend and turn up to 200ns and eventually form the stable structure until the simulation’s end. Similarly, the helices H5, H6 form stable structures in all the systems of FXR (Supplementary Figure S4). The residues of loops L: H5/H6 changes from turn or loop to coil and then bends throughout simulation in System B as compared to other systems. The loop L: H11/H12 shows the characteristic fluctuation in the different systems of FXR (Supplementary Figure S5). These residue forms a pi helix and bends in System A and B, respectively. However, in System C, the loop residues change from loop to pi helix then bend and eventually gain loop form towards the end. In System D, the residue forms the bend and then eventually regains its turn or loop form during the end of the simulation as depicted in Supplementary Figure S5. It is noticeable that this change in regions was not highlighted earlier for ‘OCA’ (Costantino et al., 2005). The secondary structure analysis indicated the presence of ‘OCA’ caused the significant conformational changes in the loops forming the binding cavity of FXR. The ‘co-activator’ binding stabilized these loops and systems showed the minimum secondary structure changes in these regions.

### Conformational Flexibility in LBD of FXR

The conformations accessed by LBD are very flexible with different binding partners. To explore it we have performed PCA analysis. The PCA reflects the collective motions of a protein during simulation (Maisuradze et al., 2009). We have shown the cumulative contribution with respect to the PC components for each system (Supplementary Figure S6A). It can be observed that overall contributory motion in System B is more than 72% of total fluctuation due to the first 10 PCs, while the top 10 PCs contribute 65, 65, and 64% of total motion respectively in the other Systems A, C, and D (Supplementary Figure S6B). This observation suggests that System B shows the highest fluctuation among the other systems as well as RMSD and RMSF plots. On the other hand, System D has shown the least fluctuation, which signifies that both ‘OCA’ and ‘co-activator’ binding stabilize the overall FXR systems. In addition, the fractional contribution plot of the top 10 PCs, the first two PCs, PC1 and PC2 appear to capture the notable variations between the systems (Supplementary Figure S6B).

**FIGURE 4:**
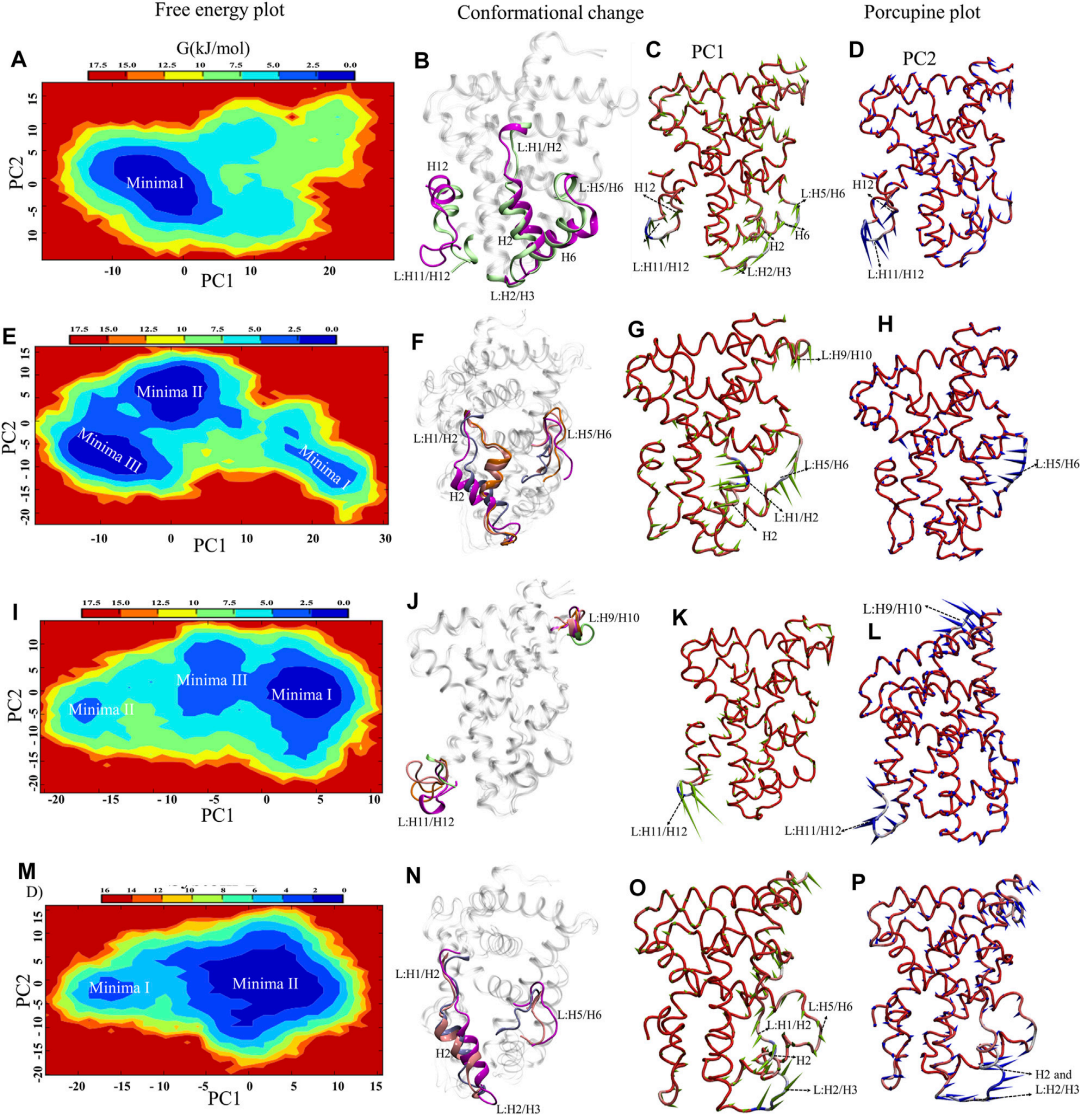
Essential Dynamics of FXR LBD systems. The panel **(A,E,I,M)** show the 2D free energy plot along PC1 and PC2 for the System A, B, C, and D, respectively. The represented minima for each system depicting the significant conformational changes with respect to crystal are shown in panel **(B,F,J,N)** for all systems. The represented minima for each system of FXR were extracted as minima 1 (magenta), minima II (pink), minima III (orange) and compared with the crystal conformation of System C (lime) and System D (ice blue). The porcupine plots represent the principal motions along the direction of PC1 and PC2. The panel **(C,G,K,O)** and **(D,H,L,P)** described the significant motion in the different systems of FXR along PC1 and PC2, respectively. The PCA component represents the atomic fluctuation of protein around its mean structure. The direction of motion is represented by an arrow, while the length of the arrow characterizes the movement strength. The protein is depicted in tube representation and coloured in blue–white-red, whereas red represents the maximum Cα displacement. The variations in the region were highlighted in the dotted arrow.

Understanding the structural dynamics of FXR complexes is important therefore, we constructed FEL along with the first two PCs as reaction coordinates in the 2D plot that reflect specific properties of the systems and measure conformational variability (Figure 4). The size and shape of the minimal energy area (basin: in blue) indicate the stability of a system. Smaller and more centralized basins suggest that the corresponding complex is more stable. Porcupine plots utilizing PC1 and PC2 are constructed in Figure 4 to indicate the locations with high atomic fluctuations and their directionality in the FXR simulated systems. System A reflects one deep basin (Minima1) (Figure 4A). These basins correspond to the conformational changes in helices H6, H12, and loops L: H2/H3, L: H5/H6, L: H11/H12 (Figure 4B) and based on the direction and magnitude of the porcupine vector, the highest fluctuation is seen in loop L: H11/H12 and anticorrelated movement to loops L: H2/H3, L: H5/H6, and helix H6 along PC1 (Figure 4C). PC2 captured the highest fluctuation in helix H12 and loop L: H11/H12 (Figure 4D). This shows that in absence of any binder, these regions constituting the binding pocket of agonist are flexible and move inward, resulting in reduced gyration and binding pocket volume as in concordance with the above-discussed sections. In System B, we observed three low-energy basins (Minima I, Minima II, and Minima III) along PC1, but the deepest is the Minima II and III (Figure 4E).

These basins correspond to the conformational changes in loops L: H1/H2, L: H2/H3, L: H5/H6, and helix H2 (Figure 4F). In porcupine plots, the helix H2 and loops L: H1/H2 and L: H5/H6 show the anticorrelated movement with each other and capture the highest fluctuation along PC1 (Figure 4G). The slight outward movement in loop L: H9/H10 was also observed along PC1. In PC2 the higher fluctuations were captured in loop L: H5/H6 (Figure 4H). The superimposition of crystal structure and the representative structures from each minimum were compared in terms of deviation from each other through RMSD analysis (Supplementary Table S4). This signifies that the binding of ‘OCA’ caused the subtle changes in these regions and significant stabilization is seen in the loop L: H11/H12 and helix H12. In System C, the FEL plot revealed three low-energy basins (Minima I, Minima II, and Minima III) along PC1, but the deepest basin was Minima I (Figure 4I). The superimposition of the structures reflects the conformational changes in loops L: H9/H10 and L: H11/H12 (Figure 4J). Despite the presence of a ‘co-activator’ in the porcupine plot of System C, it shows the highest fluctuation in the loop L: H11/H12 as compared to the other systems along PC1 (Figure 4K). The PC2 shows the fluctuation in loop L: H9/H10 higher (Figure 4L). This indicates that the binding of the ‘co-activator’ and ‘OCA’ alone causes the internal fluctuation in the FXR which is far away from the binding region of ‘OCA’ and ‘co-activator’. In System D, we observed the two basins (Minima I and Minima II), out of which the minima II is a deep basin along PC2 (Figure 4M). Upon comparing conformation changes, we found the significant changes in helix H2 and loops L: H1/H2, L: H2/H3, and L: H5/H6 in System D (Figure 4N). The porcupine plot for PC1 shows inward movements in the helix H2, loops L: H2/H3, and L: H2/H3 shows the anti-correlated motion with the loop L: H5/H6 (Figure 4O). PC2 captured the highest fluctuation in the helix H2 and loop L: H2/H3 (Figure 4P).

In System D the represented structure does not deviate much from the crystal structure as compared to System B (Supplementary Table S4). In general, the binding of the agonist to FXR the helix H12 adopts the conformation and stabilizes ‘co-activator’ peptide binding (Mi et al., 2003). However, the ‘co-activator’ can bind with the FXR in the absence of ‘OCA’ with the weaker binding affinity and causes more fluctuation in loop L: H11/H12 which can be seen in System C compared to other systems. The ‘OCA’ and ‘co-activator’ binding alone as in Systems B and C bring out the subtle changes in the helix H2 and loop regions of the LBD of FXR, while binding of both to the FXR stabilizes the system.

### Binding Site Analysis of ‘OCA’ With/Without ‘Co-Activator’

**FIGURE 5:**
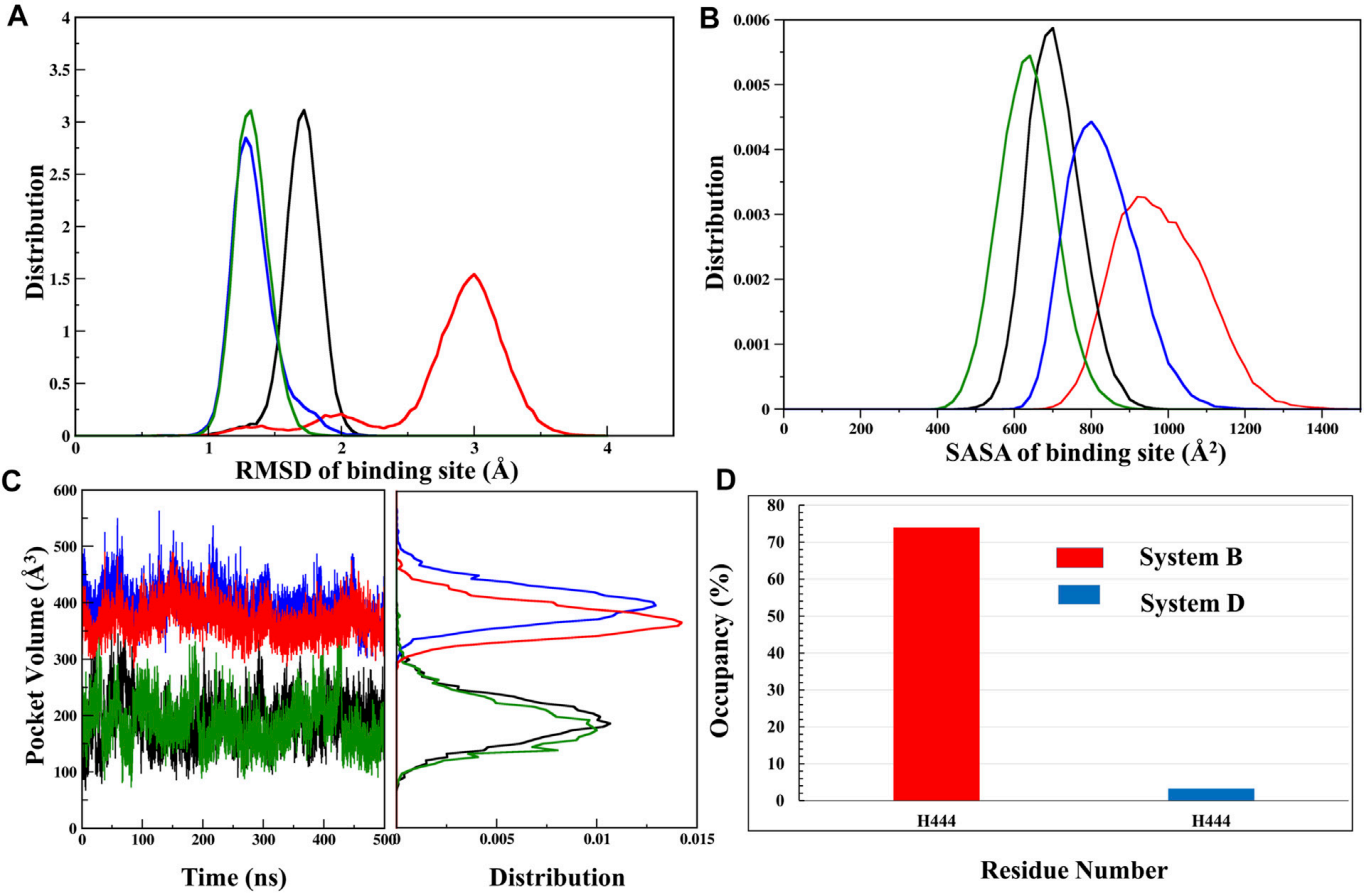
Analysis of binding site. **(A)** The RMSD distribution plot, **(B)** SASA distribution, and **(C)** pocket volume for the binding site of System A (black), System B (red), System C (green), and System D (blue) were shown throughout the MD trajectory. **(D)** The occupancy plot of residue H444 forming the water bridge interaction (wHB) with the ‘OCA’ is shown in Systems B and D.

FXR’s LBD comprises a hydrophobic pocket leading to lipophilic molecules such as BAs. As per the previously described results, it is noticed that the binding ‘OCA’ and ‘co-activator’ causes the conformational changes in the LBD of FXR. The binding site of FXR is known to have considerable flexibility to accommodate the various chemotypes (Massafra et al., 2018). To discern this dynamic of the binding site of ‘OCA’, we have explored the binding site RMSD, SASA, and pocket volume throughout the MD simulation. The backbone RMSD distribution plot for the binding site of ‘OCA’ alone in System B has a wider distribution with multiple peaks with most of the population at nearly 3.0 Å as compared to other simulated systems (Figure 5A, Supplementary Table S5). This confirms the flexible nature of the FXR pocket. The SASA distribution plot of the binding site of System B is found to be more solvent-exposed than the other three systems (Figure 5B, Supplementary Table S5), which signify that the binding of ‘co-activator’ make the pocket more stable. Further, it is also seen that the presence of ‘OCA’ and ‘co-activator’ increased the pocket volume which significantly reduced in System A indicating that the agonist increases the pocket volume of LBD of FXR (Figure 5C, Supplementary Table S5).

Upon multiple sequence alignment of rat and human FXR sequences, the similarity and identity are 96 and 92%, respectively, (we have shown similarity here) however, binding site residues are 100% conserved in rats and humans within 4.0 Å of from the center of ‘OCA’ (Supplementary Figure S7A) (Downes et al., 2003; Mi et al., 2003). The helices and loops which are involved in the binding are highlighted in Supplementary Figure S7B. The ‘OCA’ binding is mediated by the 25 residues mainly involving the hydrophobic interaction, among which only 5 residues, R328, S329, Y358, Y366, and H444, are involved in the establishment of the HBs with ‘OCA’ (Supplementary Figure S7C and Table 1).

**TABLE 1:** Interaction analysis of the FXR complex system with ‘OCA’ within the 4.0 Å area of the pocket.

Helices involved	Types of interaction	Residue Number
L:H1/H2	Hydrophobic	M^262^
H3	Hydrophobic	L^284^, M^287^, A^288^
	Polar	H^291^
H5	H-bond	S^329^, R^328^,
	Hydrophobic	M^325^, F^326^, I^283^, I^332^, F^333^
H6	Hydrophobic	L^345^, I^349^
L:H6/H7	Hydrophobic	I^354^
H7	H-bond	Y^358^, Y^366^
	Hydrophobic	I^359^, M^362^, F^363^
H11	H-bond	H^444^
	Hydrophobic	M^447^
L:H11/H12	Hydrophobic	W^451^
H12	Hydrophobic	W^466^

**FIGURE 6:**
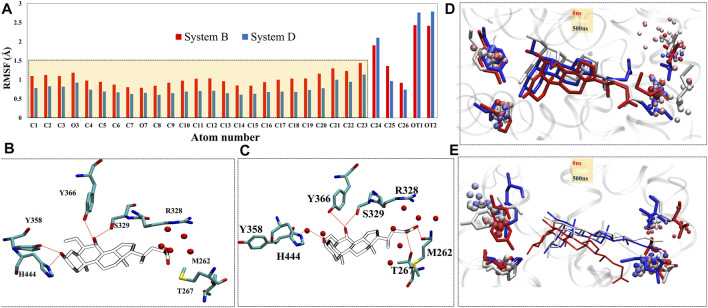
The change in the residual positioning of the LBP (ligand binding pocket) of FXR. **(A)** The ligand RMSF plot for System B (red) and System D (blue) highlighting the difference in their values. The atomic fluctuations in the head and core regions of ‘OCA’ are higher in System B than System D. **(B,C)** the panels represent HB interaction between the FXR residues and ‘OCA’ for Systems D and B, respectively. The red dotted line is the distance between the ‘OCA’ and the residue of the FXR protein. The panels **(D,E)** represent the conformational sampling for these residues in Systems D and B during MD simulation shown in time step coloring method. The representatives of initial state (red: 0 ns), intermediate state (white: 250 ns) and final state (blue: 500 ns) are shown in licorice representation while the beads representation shows the sampling of these residues throughout the trajectory by the stride of 1,000 frames at equal interval.

Upon superimposition of the binding pocket of Systems C and D, there were significant conformational changes were observed in residues M262, M287, M325, F326, R328, S329, F333, Y358, Y366, M447, and W451 in System D that are responsible to accommodate ‘OCA’ in the binding pocket of FXR (Supplementary Figure S7D). The changes in configurations of the HBs forming residues in both Systems C and D are shown in Supplementary Figure S7E. We have divided the 2D structure of ‘OCA’ (Figure 1A) to explore the residue-wise contribution, marked as three main regions, the head includes only one OH group, core (steroidal rings), and tail (carboxyl group). In the crystal (System D), the head moiety was surrounded by residues Y358, H444, M447; core region is near to residues I283, L284, V322, M325, F326, S329, F333, I345, I349, I354, I359, M362, F363, Y366, W451, and Y466; while the tail region is lined by residues M262, M287, A288, H291, R328 and I332 (Supplementary Figure S7D). The RMSF plot for ‘OCA’ in Systems B and D showed significant fluctuation in its different functional groups. Although both follow the same pattern, ‘OCA’ experienced more atomic fluctuation (atom 1–23) in System B than System D (Figure 6A).

Furthermore, the comparison of the binding site of the representative structures from the MD simulations sheds light on important residue displacements which are crucial for the binding phenomenon (Figures 6B,C). In System D, we observed that ‘OCA’ retained the HB interaction with the residues S329 (91.92%), Y358 (76%), Y366 (88.75%), and H444 (88.16%) and lost the interaction with residue R328 with respect to the crystal structure (Figure 6A, Table 2). However, in the absence of the ‘co-activator’, the ‘OCA’ lost its interaction with the residue H444 (17.34%), Y358, and R328 in System B. It gained water-mediated interaction (wHB) with the residue H444 with the occupancy of 74% as compared to System D (Figures 5D, 6C and Table 2). However, in System B the ‘OCA’ form the HB with the residues S329 (97.75%), and Y366 (92.29%) during the simulation (Figure 6C and Table 2). During MD, we found that the new residues P263, Q264, and T267 surround the tail region of the ‘OCA’ in System B, whereas in System D, the residue I294 is found in the vicinity of ‘OCA’ (Supplementary Figures S8A, S8B), which is not yet reported in previous FXR based studies This is due to the significant fluctuation in the helix H2, loop L: H1/H2 of System B than the System D. The interaction between ‘OCA’ and residues M262 and T267 possibly transient, however, seem important for their movements between stable states. We have also observed the time-line conformational changes in the interacting residues M262, T267, Y358, and H444 in both the systems (Figures 6D,E). In the case of System D, we observe the least changes in the conformation of the residues Y358, H444, and in ‘OCA’ as compared to System B, therefore form the stable interaction with it (Figure 6D). Both the residues are cryptic in nature as their interactions were missing in the initial state but came into light at intermediate state and eventually got stabilized (Figure 6D). In System B the conformational changes in the ‘OCA’ and the residue Y358 is more from the initial state which causes the loss of interaction between the residue Y358 and the ‘OCA’ and gain the transient interaction with the residues M262 and T267 (Figure 6E). The changes in the conformation of ‘OCA’ give a place for water mediate wHB interaction with the residue H444 and stabilize it in the pocket of System B. HB trajectories depicting time-dependent bond distance variations are illustrated in Systems B and D (Supplementary Figures S8C, S8D). As we observed the ligand and pocket flexibility for FXR, we speculated next about changes in the torsion angle distribution of the ‘OCA’ (tail region) in Systems B, D, and E, (Supplementary Figures S9A, S9B, see the details in supplementary results Section 3.1). Although the ‘OCA’ tail region is free to move in System D but unable to form the bond with residues M262 and T267, due to stable core region (1–23) interaction (Figure 6A). This indicates that in the presence of protein and ‘co-activator’, the ‘OCA’ behave differently and these differences in angle play a certain role in the conformational diversity of ligand ‘OCA’ (Supplementary Figures S9A, S9B). This mainly provides insights into the conformational strain undergone to maintain the protein-bound conformation.

**TABLE 2:** HB Occupancy (cut-off > 50%) of the interacting residues with ‘OCA’ in both systems B and D.

System B				
Donor	Donor atom	Acceptor	Acceptor atom	Occupancy
Y366	OH	‘OCA’	O7	97.75%
‘OCA’	O7	S329	OG	92.29%
‘OCA’	O3	H444	ND1	17.34%
**System D**				
**Donor**	**Donor atom**	**Acceptor**	**Acceptor atom**	**Occupancy**
‘OCA’	O7	S329	OG	91.92%
Y366	OH	‘OCA’	O7	88.75%
‘OCA’	O3	H444	ND1	88.16%
‘OCA’	O3	Y358	OH	76%

### Role of Cation–*π* Interactions Between the Residues H444 and W466 (Activation Trigger Zone)

**FIGURE 7:**
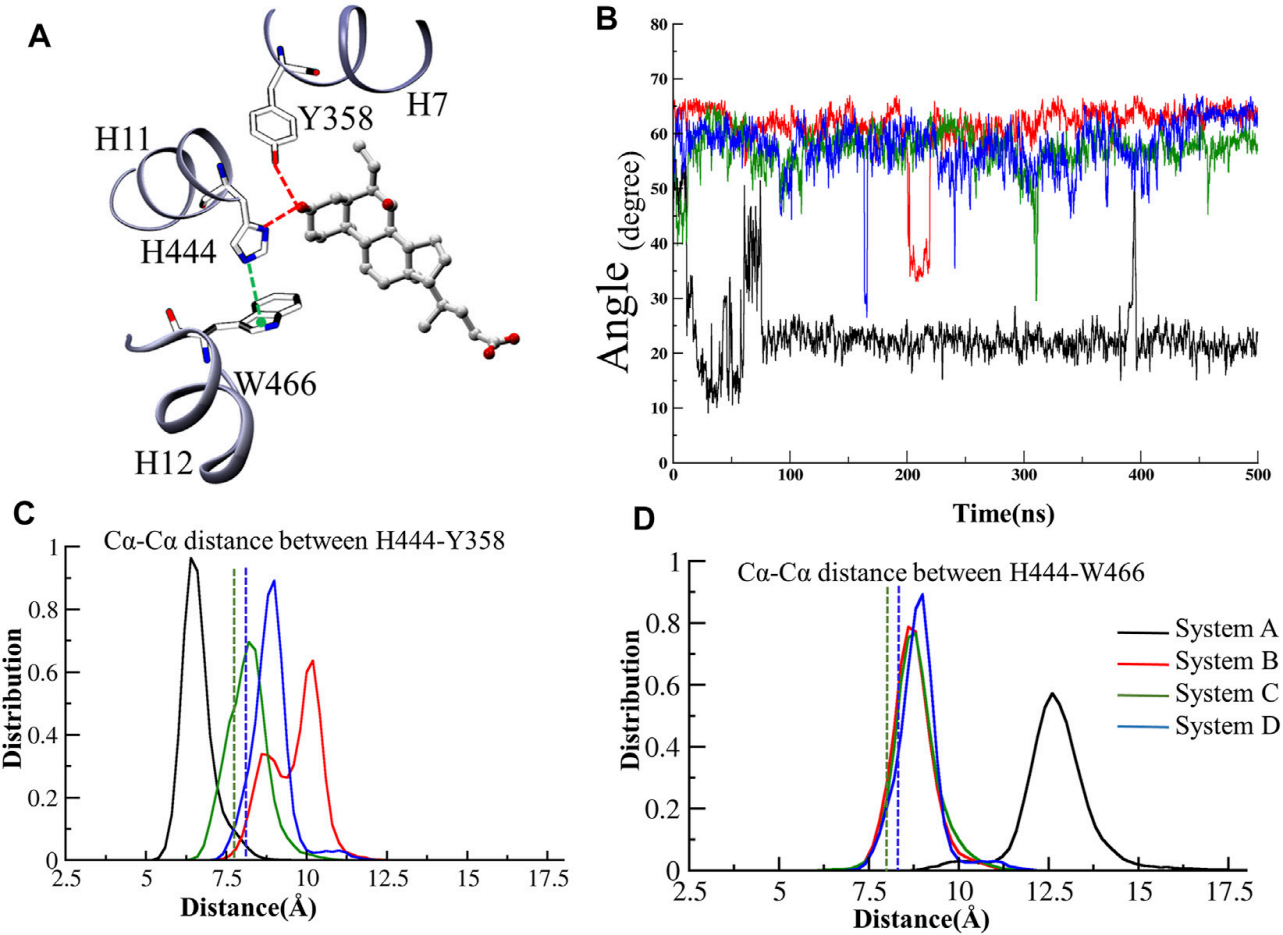
The activation trigger zone analysis. **(A)** The view of the “activation trigger” zone in the FXR structure involves the interaction between a tyrosine-histidine-tryptophan (Y358/H444/W466) triad and the ring A of the steroid bile acid backbone. The HB between the Y358 and H444 with the ‘OCA’ is shown in red dotted line. The cation-pi interaction between the NE@H444 (H10-11) atom with the center of indole ring of residue W466 (H12) is shown in green dotted line. **(B)** The time series of angle between the atoms of CD1＠W466-CE1＠H444-CH2＠W466 is depicted. The distribution plot for the distance between Cα atoms of **(C)** residues of H444 and W466 **(D)** residues Y358 and H444, throughout MD simulations. The green and blue dotted lines represent the Cα distances in the crystal structures of System C and D, respectively.

The stabilization of FXR in active conformation is based upon the interaction established between an aromatic triad tyrosine-histidine-tryptophan (Y358/H444/W466) i.e. called the (“activation trigger”) and the ring A of the ‘OCA’ (Figure 7A) (Gioiello et al., 2014; Massafra et al., 2018). It involves the HB interaction between the residues Y358 (H7) and H444 (H11) with the ‘OCA’ and the Cation–*π* interaction between the NE@H444 atom with the center of the indole ring of residue W466 (H12) shown in Figure 7A. It is also known that active conformation of the LBD requires the stability of loop L: H11/H12 than helix H12 which is achieved by the physical constraint in residue H444 (Costantino et al., 2005). The HBs formed between the 3-OH group of ‘OCA’s and the residue H444 and Y358. These interactions restrict the mobility of residue H444 and stabilize the trigger zone. The loss of their interaction would remove the necessary support for helix H12 in its active position (Mi et al., 2003). As we have discussed above, the interaction between the residues Y358 and H444 with ‘OCA’ is more stable in System D than B (Figure 7B). We also found the least fluctuation in the loop L:H11/H12 and helix H12 in System D than the other systems. Secondly, cation–*π* interactions between the indole ring of the residue W466 and NE2 atom on perpendicularly oriented residue H444 have been known to stabilize the helix H12 (Mi et al., 2003). This is the T-shaped conformation where the two planes are perpendicular, and the angle fluctuates between 45° and 145° (Khandelia and Kaznessis, 2007). To calculate cation–*π* interaction, throughout the dynamics, we calculate the angle between the atoms of CD1@W466-CE1@H444-CH2@W466. We also computed the Cα distance between the H444:W466 and H444:Y358 residues (Figure 7B). We noticed that the angle in all three Systems B, C, and D fluctuated within a range of 45°–75° during the simulation. In System A, the distance between these atoms increases during the simulation due to which the angle decreases and fails to maintain the required criteria for the angle formation (Figure 7B). This signifies that the angle between the residue H444 and W466 is stable in the presence of both ‘OCA’ and ‘co-activator’ in comparison to the APO form of FXR.

We observe that the distribution plot for Cα distance between the residues Y358 and H444 showed the distance is higher in Systems B and D than in Systems C and A (Figure 7C). However, the distance between the residues H444 and W466 is substantially more observed in System A as compared to other systems (Figure 7D). This indicates the binding of ‘OCA’ and ‘co-activator’ causing the significant conformational changes in these residues as binding decreased the distance between the residues H444 and W466 and stabilized the cation–*π* interaction in Systems B, C, and D than System A (Figure 7D). The overall analysis suggested that the binding of both ‘co-activator’ and ‘OCA’ to the FXR is necessary for the increased binding affinity. The HB distance pattern between the residues Y358 and H444 with ‘OCA’ as described above and observed in the crystal structure is maintained during the simulation in System D only and not achieved in Systems B or C.

### ‘Co-Activator’ Binding Site Analysis is Essential to Achieve Activation State of FXR

**FIGURE 8:**
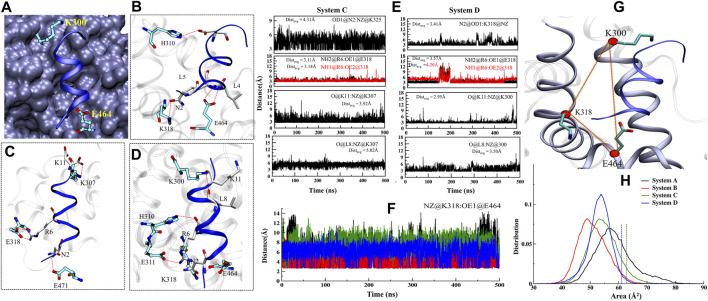
Dynamics analysis of the ‘co-activator’ binding pocket. **(A)** The view of “charge clamp” K300/307 (H3) and E464/471 (H12) in the stabilization of AF2 (blue) in structure and interacts with the ‘co-activator’. **(B)** The interacting residues in the binding site of the ‘co-activator’ in FXR crystal. **(C,D)** The interacting residues of the FXR stable MD state of System C and D. **(E)** The depiction of the HB durance throughout MD simulation. **(F)** The time series plot of distance between the NZ@K318 with OE2@E464 throughout MD simulations. **(G)** The panel represents the area between the residues K300, K318 and E464. Beads (red color) correspond to the Cα atom of each residue. **(H)** Distribution of the area defined by the Cα atoms of the residues throughout dynamics. The green and blue dotted lines indicate the area in the crystal structures of Systems C and D.

The FXR’s LBD acts as a molecular switch after ligand binding, undergoing the conformational changes that result in the recruitment of the ‘co-activator’ protein by forming the “charge clamp” and a hydrophobic groove that interact with the LXXLLmotifs of ‘co-activators’ (Weikum et al., 2018; Merk et al., 2019). It is reported in the agonistic conformation of FXR, the ‘co-activator’ (LxxLL motif) is bound by “charge clamp” with residues K300 (H3) and E464 (H12) (Figure 8A) (Merk et al., 2019). Therefore, we explored the conformational residual changes which are responsible for stabilizing helix H12 throughout the dynamics. The ‘co-activator’ binding surface on FXR comprises the helices H3, H4, H5, and H12. This analysis have shown that, with the binding of agonist in FXR, the ‘co-activator’ typically forms the four HB with the residues K300, H310, E311, and E464 of the FXR (Merk et al., 2019). In System D/C, the ‘co-activator’ forms the HBs with residues K300/307, H310/317, K318/325, E464/471 and non-bonded contacts with the FXR residues Q293/300, V296/303, E297/304, F305/312, Q313/320, I314/321, L317/324, P460/467, L461/468 and E464/471 (Supplementary Figure S10A and Table 3). We have found that in System D, HB interaction between the ‘co-activator’ residues N2, L4, L5, and D10 with the FXR residues K318, E464, and H310, respectively (Figure 8B). We observe that the residue E311 is not the vicinity of the ‘co-activator’ binding site in 4.5 Å in System D (Supplementary Figure S10A and Table 3).

**TABLE 3:** Interaction analysis of FXR with peptide within 4.5 Å.

Helices involved	Types of interaction	Residue number
H3	Hydrophobic	V^296^
	Polar	Q^293^, E^297^, K^300^
H4	Hydrophobic	F^305^
H5	H-bond	K^318^
	Hydrophobic	L^317^, I^314^
	Polar	Q^313^, H^310^
H12	Hydrophobic	P^460^, L^461^
	H-bond	E^464^

Further to see the residue interaction with the ‘co-activator’, we have analyzed the stable state for Systems C and D. The residues of ‘co-activator’ N2, R6, D12 gain the interaction with FXR in terms of HBs with K325, E318, and K300 residues (Figure 8C) and non-bonded interaction with the I472, T306, I321, I469, T292, V296, V299, L302, V303, Q300, L324 and I321 in System C (Supplementary Figure S9B). In the case of System D, the residues of the ‘co-activator’ N2, R6, K11, and L8 gain the HB interaction with FXR residues K318, E464, H310, E311, and K300 (Figure 8D) and non-bonded with the residues I465, L317, Q313, I314, T299, R301, Q306, and V292 in System D (Supplementary Figure S10C). The loss of interaction was also observed between the residues L4, L5, D12 with the E464 and K300 (Figures 8C,D).

Further, we analyzed the HB distance during dynamics (Figure 8E). We noticed that the interaction between the ‘co-activator’: FXR atom O@K11: NZ@K300 is most stable throughout dynamics in System D among the other interactions (Figure 8E). While this interaction is unstable in System C. This signifies that possibly the ‘OCA’ helps to establish the interaction with the “charge clamp” residue more stable. The interaction between the O@L8: NZ@K300 in System D is more stable than System C. While the interaction between OD1@D10:NE2@H310 becomes unstable during dynamics in both Systems C and D (Supplementary Figure S9D). In System C, the interaction between the NH2@R6:OE1@E311 and NH1@R6: OE2@E311 is more stable as compared to System D. This could be the region of retaining the ‘co-activator’ in the FXR without the presence of ‘OCA’. In System D, the interaction with OD1@N2:NZ@318 is comparably stable than System C. However, the interaction O@N2:OE2@E464, N@L4: OE1@E464, and N@L5:OE2@E464 is not stable throughout the dynamics in both the systems (Supplementary Figure S9C). This is interesting to note that the “charge clamp” residues are playing an important role in the recruitment of ‘co-activator’. Therefore, we have calculated the area between the Cα atom of residues K300-E464-K318 in all systems of FXR (Figure 8G). We take CA@E464 as an anchor residue linking the K300 and K318 residues. Throughout MD simulation, we measured the region for all FXR systems to see shifts in the “charge clamp” forming residue presence and absence of ‘OCA’ and ‘co-activator’. During the simulation timescale, the conformational changes result in a dramatic expansion in the area of the clamp in System A with respect to other systems (Figure 8H). This increase in area is due to the high flexibility of the Cα atom due to the absence of the ‘co-activator’. We found, however, that the region for System D is least extended; this could be due to the presence in the FXR of both ‘OCA’ and ‘co-activator’ binding. System C also has less distribution area than Systems A and B, which supports that, in the absence of a ligand, the FXR can retain the ‘co-activator’. While this area estimation approach is not a precise procedure, it still provides tentative details on the selection of “charge clamp” residues to explore the co-activator/co-repressor binding site with or without binding of ‘OCA’. Laying down an assumption, we could propose that the agonist binding to the FXR is always required for the strong binding of the ‘co-activator’ to the FXR. From MD analysis, we found that the agonists alone or ‘co-activator’ can bind and retain in their binding site as we have achieved confident data about their binding. However, they are unable to achieve their active state.

### Per-Residue Wise Free Energy Contributions to Identify the Critical Residues in FXR

#### Binding Free Energy of ‘OCA’ and ‘Co-Activator’ in FXR

**FIGURE 9:**
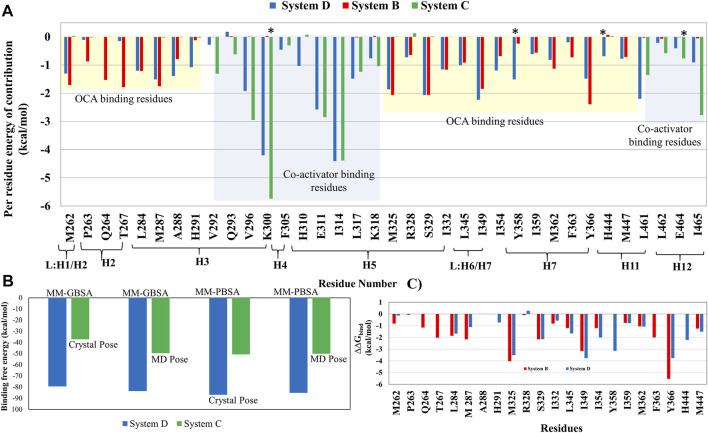
Per residue wise decomposition of the binding energy ∆G_bind_ (kcal/mol) for ‘OCA’ and ‘co-activator’. **(A)** The residues (cut off >0.5) belonging to the region in FXR are mentioned in the figure. The highlighted residues in yellow and blue color are interacting residues with the ‘OCA’ and ‘co-activator’, respectively. The residues Y358, H444, K300 and E464 in the star sign are the residues involved in “activation trigger zone” and “charge clamp”. The residues in System C are different in residue number. However, the location of the residues is the same. The residues number in Systems D/C are V292/299, Q293/300, V296/306, K300/307, F305/312, H311/317, I317/321, K318/325, L461/468, L462/469, E464/471 and I465/472. **(B)** The total energy change in the residue after alanine scanning in both System B and D. **(C)** The bar plot represents the total binding free energies of crystal poses and MD-pose in both Systems C and D.

The total binding free energies calculated as per Scheme 1 are listed in Table 4. The total ΔG_bind_ of ‘OCA’ (System B) in the absence of a ‘co-activator’ is −30.45 kcal/mol, by using the MM-PBSA method (Table 4). The total ΔG_bind_ of the ‘co-activator’ in the absence of ‘OCA’ (System C) −50.14 kcal/mol by using the MM-PBSA method. However, the total ΔG_bind_ of ‘OCA’ and ‘co-activator’ in FXR (System D) is −86.83 kcal/mol, and their MM-GBSA values are listed in Table 4. The per residue energy of each contributing residue is given in Figure 9A. Here, we noticed that the total ΔG _bind_ of ‘OCA’ is increased in the presence of a ‘co-activator’. This reflects that binding of ‘OCA’ is more energetically favored upon binding of ‘co-activator’. The higher contribution to the ΔG_bind_ in the presence and absence of ‘co-activator’ in Systems B and D is due to the difference in the ΔG_solv_ GB and ΔG_solv_ PB. Besides, the ΔG_bind_ differences in System C and D appeared due to the electrostatic interactions (ΔE_ele_) in the gas-phase and polarization contributions (ΔG_pol_), indicating that the two energetic components had remarkable effects on the binding free energy between ‘co-activator’ and FXR. The calculated total ΔG_bind_ for the crystal poses of Systems C and D is −49.54 kcal/mol and −83.65 kcal/mol, respectively by using the MM-PBSA method (Figure 9B). In terms of dynamics, it is observed that the binding free energy of System D is even improved to crystal pose, indicating the possibility of better structural fit is achieved during the simulation.

**TABLE 4:** The contribution of the binding free energy for ‘co-activator’ in Systems B, C and D. In the bracket, the standard deviation and the standard error of mean values are specified. The standard error of mean values (i.e., the standard deviation divided by the square root of the number of snapshots) to depict the precision of the MM-GBSA and MM-PBSA methods to estimate the binding free energies. The bold values indicates the total binding free energy.

Contribution	System B (‘OCA’ only)	System C (only ‘co-activator’)	System D (‘OCA’+ ‘co-activator’)
∆E_int_	0	0	0
∆E_vdW_	−53.99 (3.22 ± 0.05)	−53.54 (4.89 ± 0.08)	−108.91 (6.45 ± 0.11)
∆E_ele_	−182.24 (11.93 ± 0.22)	−209.92 (35.81 ± 0.65)	−301.84 (38.30 ± 0.60)
∆E_GB_	201.77 (11.66 ± 0.22)	221.75 (33.52 ± 0.61)	339.98 (35.39 ± 0.64)
∆E_surf_	−7.23 (0.34 ± 0.006)	−8.93 (0.57 ± 0.01)	−16.06 (0.83 ± 0.01)
∆G_gas_	−236.24 (12.75 ± 0.23)	−263.47 (36.18 ± 0.66)	−410.75 (38.84 ± 0.70)
∆G_solv GB_	194.54 (11.53 ± 0.21)	212.82 (33.33 ± 0.60)	323.92 (35.39 ± 0.64)
**∆G**_**GB**_	**−41.67 (3.45 ± 0.06)**	**−50.65 (6.22 ± 0.11)**	**−86.83 (7.86 ± 0.14)**
∆G_solv PB_	205.78 (13.85 ± 0.25)	−53.54 (4.89 ± 0.08)	325.60 (36.24 ± 0.66)
∆E_PB_	210.71 (13.83 ± 0.25)	220.06 (33.35 ± 0.60)	336.56 (36.39 ± 0.66)
∆E_n-polar_	−4.92 (0.10 ± 0.001)	6.73 (0.26 ± 0.004)	−10.96 (0.37 ± 0.006)
**∆G**_**PB**_	**−30.45 (6.63 ± 0.12)**	**−50.14 (7.95 ± 0.14)**	**−85.15 (10.90 ± 0.19)**

#### Per Residue Wise Energy Contribution in FXR-‘OCA’ Interactions

Analysis of the residue wise free energy decomposition was also carried out to analyze the individual energetic contributions of each residue involved in the stabilization of protein-ligand complexes. To understand the interactions at the atomic level, binding free energy contributions were determined for each residue for Systems B and D (Figure 9A, Supplementary Table S6). The residues having a contribution of (−0.5 kcal/mol) or above were considered *hot-spot* amino acids and were positioned to contribute most to the stability of the complex. As per the cutoff of the residues M262, P263, Q264, T267, L284, M287, A288, M325, R328, S329, I332, L345, I349, I354, I359, M362, F363, Y366, and M447 have shown high energy contribution in System B (Figure 9A). In the case of System D, the residues M262, L284, M287, A288, H291, R328, S329, M325, I332, L345, I349, I354, Y358, I359, M362, Y366, H444, and M447 has shown the highest contribution in the presence of ‘co-activator’. Most of the residues are common in both the systems except the M262, T267, L284, and F363 in System B and H291, H444, and Y358 in System D. The residues M262, Q264, T267, and S329 make remarkably high free energy contributions hence, making a considerably enormous contribution to the overall binding free energy of System B and in System D the residues M325, S329, and I349 contribute more to the overall binding energy. To determine the detailed contribution of each important residue, the binding energy was decomposed into electrostatic, VdW, solvation (polar and nonpolar), and total contribution (Supplementary Table S6). The thermodynamic profiling suggests that the electrostatic and VdW are the major contributors to the ‘OCA’ net binding. The residue R328, which forms a HB with ‘OCA’ in the crystal structure, reveals an unfavorable contribution towards the total binding free energy, as this interaction was not sustained in both the systems. In System B the ‘OCA’ also gained interaction with residues M262 and T267 which can see their higher contribution in System B than D. Thus, the thermodynamic profiling suggests that the contribution of the residue plays a major role in the binding of the ‘OCA’ to FXR. The analysis revealed that ‘OCA’ is stable and gains substantially favorable interactions with the pocket residues. However, we further perform the CAS to elucidate their impact on the binding energy of the systems.

#### Key Residue Contributions in FXR and ‘Co-Activator’ Interactions

We have calculated residue-wise decomposition to identify critical residues involved in protein -‘co-activator’ interaction in Systems C and D and take the same cut-off, shown to be above −0.5 kcal/mol (Figure 9A, Supplementary Table S7). We observed the “charge clamp” residue K300/307 and the highest contribution in total binding energy in the presence (System D) and absence of ‘OCA’ (System C) (Figure 9A). Besides this, we found the residues V292/299, V296/306, E311/318, I314/321, L317/324, K318/321, L461/468, I465/472 contributed higher to the binding energy (−1.0 kcal/mol). This indicates that the hydrophobic residues facilitate the repacking of the helix H12 as the non-polar residues V296/306, I314/321, L461/468, I465/472 contribute the above −2.0 kcal/mol to total binding energy in FXR (Figure 9A). These values indicated the possibility that the ‘co-activator’ can bind to FXR in the absence of ‘OCA’.

### Cross-Validation of Residue Wise Contribution in the Stability of ‘OCA’ *via* Computational Alanine Scanning

To accomplish the contribution of the identified residues to the total free energy, we performed computational alanine scanning. The obtained results indicate that the mutation in residues has significantly dropped the binding energy by more than −1.0 kcal/mol (cutoff) in both the complexes (Figure 9C). In residues M287, S329, M325, I349, and I354 typical in the presence and absence of a ‘co-activator’, a substantial decrease in binding energy (−2.0 kcal/mol) was observed. The residues T267 and F363 had a significant reduction in binding energy while they were interacting in presence of ‘OCA’ alone. The residues Y358 and H444 form the direct interaction with the ‘OCA’ in the presence of a ‘co-activator’ and have a significant drop in the (>−2.0 kcal/mol) binding energy. Therefore, one can infer the key *hot-spot* residues T267, M325, I349, Y358, S329, F363, Y366, and H444 important for the ‘OCA’ recognition mechanism in FXR. The presence of a ‘co-activator’ establishes a stable interaction of ‘OCA’ with the FXR, which is responsible for the activation mechanism of FXR.

## Discussion

To unveil the binding event of ‘OCA’ and ‘co-activator’ at its functional level, we have evaluated the four systems of FXR and the possible mechanism for activation at the molecular level by using the triplicates of MD simulations.

The conformational change of FXR–LBD in response to different molecular binding, such as agonist and partial agonist is significant for recruitment of ‘co-activator’ protein and release of co-repressor. Several studies have been proposed to analyze the interactions of FXR with agonist, antagonist, or with and without co-through molecular modeling (Costantino et al., 2005; Meyer et al., 2005; Zhang et al., 2006; Di Leva et al., 2013). However, here, we tried to speculate how the active state of FXR has been obtained at the structural conformational level and how this internal motion helps to modulate the specific region, which provides the specific platform for activation of FXR in synergy between the binding sites of ‘co-activator’ and agonist.

### Perturbed Mobility of Loop L: H11/H12 is Essential for the Activation of LBD

During the simulation, the systems with the binding partner as a ‘co-activator’ alone and with ‘OCA’ remained remarkably stable. The compactness in the system without ‘co-activator’ and ‘OCA’ signifies that the binding of both disturbs the internal dynamic behavior of FXR (Figure 2B). Compared to Systems A and C, the binding of both ‘OCA’ alone and with a ‘co-activator’ had significantly decreased the RMS fluctuation in loop L: H11/H12 and helix H12 (Figures 3G,H). This indicates that the binding of ‘OCA’ is essential for the conformational changes at helix H12. The DCCM map revealed that the helix H3 shows the correlated motion with loop L: H11/H12 and helix H12 in presence of ‘OCA’ (System B) and both ‘OCA’ and ‘co-activator’ (System D) (Supplementary Figure S11, see the details in supplementary results Section 3.2). The enhanced correlation in the agonistic conformation comes from the increased stability of the loop L: H11/H12 and the helix H12 whose stability is critical to maintain the agonistic conformation. This is in concordance with RMSF results as well where the fluctuations in helix H12 and loop L: H11/H12 get reduced in the presence of ‘OCA’ and ‘co-activator’ both. The essential dynamics also reveal that the presence of ‘OCA’ and ‘co-activator’ maintain the stability in loop L: H11/H12, which is not found in the presence of either ‘co-activator’ or agonist alone (Figures 4J–L). Since the loop L: H11/H12 controls the flexibility of helix H12 and a critical determinant for its orientation (Costantino et al., 2005; Merk et al., 2019). Therefore, the study of this region is essential to understand the mechanism of different types of ligand binding in FXR.

### Flexibility Allows Reaching the Activate State Conformation by Modulating *via* ‘OCA’ at Agonist and ‘Co-Activator’ Binding Sites

We observed that ‘OCA’ and ‘co-activator’ are substantially stable according to the different analyses. The binding of both either only ‘OCA’ alone or with a ‘co-activator’ caused the higher fluctuation in helix H2 and loops between L: H1/H2, L: H2/H3, L: H5/H6, and L: H9/H10 regions which signify the flexible nature of LBD of FXR. However, the ‘co-activator’ binding stabilizes the helix H2 and loop L: H2/H3 with ‘OCA’. This is in concordance with secondary structure analysis results where the conformational flexibility of these regions is higher in the presence of ‘OCA’ (Supplementary Figures S3–S5). Furthermore, we found that in the presence of ‘OCA’ there is an anticorrelated movement in the helix H2 and loops L: H1/H2, L: H5/H6 region of FXR, which is absent in the presence of ‘co-activator’ alone. These changes in loop conformation account for the increase in SASA and RMSD values of the binding site in the presence of ‘OCA’ alone which stabilized in the presence of a ‘co-activator’. The volume of the LBD pocket was shown to vary significantly during the simulation and revealed the flexible nature of the pocket of FXR. Combined with RMSF analysis of the ‘OCA’, one possible explanation is that the core region of the ‘OCA’ is stable in the presence of a ‘co-activator’, and just increased the fluctuation at the tail region of ‘OCA’. The binding of ‘OCA’ induced the significant expansion of the LBD, which is why for its increased pocket volume.

### Changes in Hydrogen-Bond Network Upon ‘OCA’ and ‘Co-Activator’ Binding

The HBs between a protein and ligands provides directionality and specificity of interaction, an important aspect for molecular recognition. The considerable changes were observed in the binding pocket of FXR in the presence of ‘OCA’ and ‘co-activator’. In our study, the residues S329 and Y366 are noticed to be common in the presence and absence of a ‘co-activator’ (occupancy >50%) Table 2. It is surprising that, in the absence of a ‘co-activator’, the Y358 and H444 residues lose their contact completely with ‘OCA’ during the simulation and stay in the pocket. This is attributable to the establishment of the stable association of wHB with H444 residues (occupancy >70%) (Figure 5D). In the crystal structure of ‘OCA’ the HB is formed with R328, which is lost during the simulation, and it’s tail region also forms the transient interaction with the M262 and T267 residues, which are absent in the presence of a ‘co-activator’. The ‘co-activator’ interacting residues with FXR is also more stable in System D than System C (Figure 8E). Free energy per residue decomposition and alanine scanning confirm the contribution of the key residues maximum in System D. However, the comparable energy contribution from Systems B and C indicates that achieving the critical orientation is important for the individual residue.

### The Role of “Activation Trigger Zone” and “Charge Clamp” in Stability of Helix H12

We noted in the presence of ‘OCA’ and ‘co-activator’ the persistent formation of the angle between the residues Y358, H444, and W466; while in apo these residues are unable to form this angle during dynamics. The cα distance between them is also found to be stable in presence of the ‘OCA’ and ‘co-activator’ compared to APO. Costantino et al. reported that the HB interaction between the ‘OCA’ and residues Y358 and H444 is not sufficient to stabilize the helix H12 since it is already in an active conformation in the absence of ‘OCA’, and there is stable interaction between the residues H444 and W466 (Costantino et al., 2005), and the same was achieved in our studies. Interestingly, we found that in the presence of ‘OCA’, the wHB played an important role in restricting the movement of residue H444 in the LBP of FXR and stabilizing the helix H12 conformation. The residue of “charge clamp” (K300) formed the stable interaction with the residues L8 and K11 of the ‘co-activator’ in System D than C, which confirms that the binding of ‘OCA’ enhances the association of the ‘co-activator’ with FXR (Figure 8E). It has been also reported that in the absence of an agonist, the ‘co-activator’ is inaccessible to FXR due to the formation of a salt-bridge between the residues K318 and E464 (Costantino et al., 2005). But in our result, we confirm that there is no stable salt bridge formation between the residues K318 and E464 during the simulation (Figure 8F). Henceforth, the FXR can be bound with the ‘co-activator’ in the absence of ‘OCA’ but the stability of loop L: H11/H12 is necessary for stabilizing helix H12, which is not consistent.

### Key Feature Determining the Binding of ‘OCA’

The quantitative characterization of binding free energies of specific residues in protein−ligand binding is critical as these residues are capable of modulating the internal wiring from function to non-function state and vice-versa. Here, we have elucidated the key interaction captured by the ‘OCA’ in the presence and absence of a ‘co-activator’. Binding free energy calculations suggest the ‘OCA’ affinity is highest with ‘co-activator’ binding to FXR. Based on the results of per residue binding free energy decomposition, we can observe that the number of residues stabilizing the complex as well as the energetic weight of each interaction contributes to the main differences in the total binding free energy. ‘OCA’ is well stabilized in the presence of a ‘co-activator’, as a result of forming similar interactions with comparable per residue-free energy contributions to the total binding free energy.

The residues L284, M325, S329, I345, I349, I354, I359, M362, F363, Y366, and W466 occupied the core region of ‘OCA’ have shown the higher contribution towards the binding energy alone and with the presence of a ‘co-activator’. The residues P263, Q264, T267 near the tail region of ‘OCA’ which gain interaction during dynamics have a higher contribution in ‘OCA’ at System B. This signifies the ‘OCA’ stability in the pocket of FXR in absence of a ‘co-activator’. As reported earlier, the 6α-ethyl group (head region) in ‘OCA’ binds into the hydrophobic cavity that exists between the side chains of residues I359, F363, and Y366 increases the affinity of ‘OCA’ (Pellicciari et al., 2002). We also found that these residues show a substantial contribution towards the total binding energy in the presence of ‘OCA’ and confirmed the important role of residues in the molecular recognition of ‘OCA’ in the FXR pocket. In the presence of a ‘co-activator’, the residues Y358 and H444 contribution is higher along with the other residues. However, we did not find any contribution from the residue F363 in presence of a ‘co-activator’. This means that ‘OCA’ governs the stability in the FXR pocket in the presence and absence of ‘co-activator’ differently and only ‘co-activator’ binding is required for agonist discovery.

### Key Feature Determining the Binding of ‘Co-Activator’

The thermodynamic profiling suggests that the electrostatic and VdW are the major contributors in the net binding of the ‘co-activator’ in the presence and absence of ‘OCA’. The residues of the “charge clamp” formation play an important role in the co-activator-interacting surface, exist in many NRs, and can stabilize their active conformations (Nolte et al., 1998; Wang et al., 2017). In our study, we also noticed the stable interaction with the “charge clamp” residue K300/307 and the highest contribution in net binding energy (Figure 9A) in Systems D and C. However, the lowest contribution came from the residue E464. Furthermore, Merk et al. had studied that the unliganded form of FXR was also able to recruit the ‘co-activator’ (Merk et al., 2019). This result also suggested that the contribution of residue is substantial in absence of ‘OCA’ and the ‘co-activator’ can bind with the FXR.

### Binding *Hot Spot* for ‘OCA’

Based on the consistent information of interaction analysis and CAS, a significant drop in the binding energy more than −2.0 kcal/mol were noticed in the residues T267, M287, S329, M325, I349, I354, Y358, F363, and H444 in the presence and absence of ‘co-activator’ (Figure 9C). Overall, these data indicate that to achieve a specific active state of FXR certain residue orientation must be targeted to activate the FXR agonism. Overall, conformational, and residual synergy has been observed between agonist and ‘co-activator’ binding sites. The RMSD, SASA plots, and distance variation between residues H444-Y358 and residues H444-W466 reflect the binder-dependent dynamical adjustment at the architectures of binding sites that correlated well with thermodynamic outcomes. This indicates that both sites and their residual position must be considered to improve and discover modulators.

## Conclusion

The complete activation of FXR by OCA blocks the BAs synthesis and hinders metabolic cholesterol degradation. As a result, studying FXR conformational changes in the presence and absence of ‘OCA’ and ‘co-activator’ seems essential to explore. Here, we have leveraged our understanding in molecular association between binding sites of ‘co-activator’ and agonist using detailed dynamics analysis of four comparable systems.

MD simulations divulged profound shifts of the different helices in the FXR systems. Our work mainly explores the binding mechanism of ‘OCA’ in FXR. Further, correlation analysis reveals that a global network of the correlated motions exists in the FXR, whose components include all regions identified so far to be critical for the binding of ‘OCA’. The increase in ΔG_bind_ energy suggested that the presence of a ‘co-activator’ increased the binding affinity of ‘OCA’ with FXR. The ∆E_ele_ energy is more favorable in presence of both ‘OCA’ and ‘co-activator’ alone. However, the ∆E_ele_ is most favorable in binding ‘OCA’ with a ‘co-activator’. The CAS analysis further confirms the individual contribution to the total binding energy. Our results pointed to residues M262, T267, M287, M325, S329, I349, Y358, Y366, and H444, in an FXR, found to be more crucial for binding of ‘OCA’. The agonist and ‘co-activator’ binding with FXR, an activation state in which the loop L: H11/H12 and helix H12 are completely stabilized and the interactions remain intact to keep the architecture of their binding pockets. The lack of ‘OCA’ in the binding pocket of FXR makes loop L: H11/H12 extremely unstable. However, the ‘co-activator’ binds to FXR. It implies that to keep this loop stable, the ‘OCA’ binding is necessary. In the absence of a ‘co-activator’, the ‘OCA’ loses its significant interaction with the residues Y358 and H444 which is necessary for the “activation trigger” in FXR. Thereby improving our understanding of ‘OCA’ and ‘co-activator’ binding sites in FXR provide a promising basis for future agonist discovery. Overall, the conformational characterization and dynamical synergy between the binding sites and residues of the ‘co-activator’ and agonist could be explore further for better mechanistic understanding.

## Data Availability

The raw data supporting the conclusions of this article will be made available by the authors, without undue reservation.

## Abbreviations

FXR, Farnesoid X receptor; MD, molecular dynamics; AF1, activation function 1; DBD, DNA-binding domain; LBD, ligand-binding domain; 6-ECDCA, 6-ethylchenodeoxycholic acid; MM–GBSA, molecular mechanics–generalized born surface area; GAFF, general amber force field; MM, molecular mechanics; PCA, principal component analysis; FEL, Free energy landscape; CAS, computational alanine scanning.
