# Supplementary material for: Conformational Characterization of the Co-Activator Binding Site Revealed the Mechanism to Achieve the Bioactive State of FXR
Source: Front Mol Biosci. 2021 Aug 31;8:658312. doi: 10.3389/fmolb.2021.658312 (PMC8439381; doi:10.3389/fmolb.2021.658312)
Supplement: Supplementary file 1 [file DataSheet1.pdf]

Supporting Information

Conformational characterization of the co-activator binding site revealed the mechanism to achieve the bioactive state of FXR

Anita Kumari[1,2], Lovika Mittal[1], Mitul Srivastava[1], Dharam Pal Pathak[2,3], Shailendra Asthana[1*]

[1]Translational Health Science and Technology Institute (THSTI), Faridabad, Haryana, India

[2]Delhi Pharmaceutical Sciences and Research University (DPSRU), New Delhi, Delhi, India

[3]Delhi Institute of Pharmaceutical Sciences and Research (DIPSAR), New Delhi, Delhi, India

*To whom correspondence should be addressed:

Dr. Shailendra Asthana

Principal Scientist

Translational Health Science and Technology Institute (THSTI),

NCR Biotech Science Cluster, 3rd Milestone

Faridabad – Gurgaon Expressway, Haryana-121001, India

E-Mail: sasthana@thsti.res.in

1. Supplementary Tables

Table S1: Summary of all FXR systems.

**Type**	**Systems**	**PDB IDs**	**species**	**Resolution(Å)**	**Binder type**
FXR without ligand and coactivator	System A	5Q0K	Human	1.8	-
FXR with co-activator (GRIP-1 peptide /co-activator)	System C	5Q0K	Human		ENALLRYLLDKD
FXR with 'OCA'	System B	1OSV	Rat	2.5	'OCA'
FXR with 'OCA' and co-activator	System D	1OSV	Rat		ENALLRYLLDKD
Only 'OCA' with water	System E	-	-	-	'OCA'

Table S2: Summary for the FXR systems for MD simulation.

Systems	MD length (ns)	Solute atoms	ions	water molecules	Total atoms
System A	500 x 3	3735	9	29766	33510
System B	500 x 3	3831	10	32691	36532
System C	500 x 3	3944	10	32127	36081
System D	500 x 3	4040	11	32421	36472
System E	500	73	0	2713	2887

Table S3: The average values for the calculated parameters for all the systems of FXR obtained after 500 ns MD simulations.

**Systems**	**$RMSF_{avg}$ (Å)**	**Radius of $Gyration_{avg}$ (Å)**
System A	0.99	17.73
System B	1.18	18.12

System C	0.97	18.11
System D	1.01	18.19

Table S4: The difference in the represented structure of FXR and ‘OCA’ from their crystal.

**FXR Systems**	**Representative structures**	**$RMSD_{avg}$ (Å)**	
		Protein	‘OCA’
System A	Minima 1	1.96	-
System B	Minima1	1.88	1.63
	Minima II	2.29	2.01
	Minima III	2.34	2.23
System C	Minima I	1.40	-
	Minima II	1.89	-
	Minima III	1.42	-
System D	Minima I	2.04	3.08
	Minima II	1.31	1.87

Table S5: The summary of the average values for parameter of the binding site of FXR in all systems.

**FXR Systems**	**$RMSD_{avg}$ (Å)**	**$SASA_{avg}$ (Å^2)**	**Pocket $volume_{avg}$ (Å^3)**
System A	1.68	694.51	190.94
System B	2.80	971.14	369.75
System C	1.30	624.87	184.11

System D	1.32	818.70	398.10

Table S6: Per residue decomposition of the binding energy for 'OCA' in System B. The table contains the decomposition of calculated ΔG_{bind} (kcal/mol) on per residue basis into VdW, electrostatic, polar, and non-polar solvation energy component.

System B (Residue number)	Vander Waals	Electrostatic	Polar Solvation	Non-Polar Solv.	TOTAL
	ΔE_{vdw}	ΔE_{ele}	$\Delta G_{sol\ GB}$	$\Delta G_{sol\text{-}np}$	ΔH_{GB}
M262	-1.024	-5.12	4.63	-0.20	-1.71
P263	-0.547	-2.40	2.10	-0.02	-0.87
Q264	-0.815	-5.40	4.79	-0.10	-1.53
T267	-0.965	-3.07	2.38	-0.12	-1.78
L284	-1.262	-0.62	0.81	-0.13	-1.21
M287	-1.92	-1.10	1.48	-0.21	-1.75
A288	-0.66	-0.26	0.18	-0.05	-0.79
H291	-0.35	-0.94	1.19	-0.02	-0.12
V292	-0.04	0.11	-0.08	0	-0.01
Q293	-0.01	0.34	-0.30	0	0.03
V296	0.00	0.22	-0.21	0	0.01
K300	0.00	7.70	-7.67	0	0.03
F305	0.00	0.09	-0.09	0	0.00
H310	0.00	-0.10	0.10	0	0.00
E311	0.00	-5.28	5.27	0	-0.01
I314	0.00	-0.20	0.20	0	0.00
L317	0.00	-0.20	0.19	0	-0.02
K318	0.00	6.54	-6.50	0	0.04
M325	-2.36	-2.65	3.26	-0.32	-2.06
R328	-0.91	18.62	-18.22	-0.14	-0.65
S329	-1.42	-3.20	2.70	-0.14	-2.06
I332	-0.91	1.09	-1.14	-0.21	-1.16
I345	-0.82	0.62	-0.61	-0.11	-0.91
I349	-1.63	0.40	-0.41	-0.20	-1.84
I354	-0.57	0.35	-0.40	-0.07	-0.68
Y358	-0.53	-0.28	0.61	-0.04	-0.24
I359	-0.73	-0.26	0.46	-0.03	-0.56

M362	-0.98	0.36	-0.44	-0.07	-1.13
F363	-1.36	0.25	0.52	-0.13	-0.72
Y366	-0.91	-2.07	0.74	-0.15	-2.39
H444	-0.59	-0.89	1.62	-0.08	0.07
M447	-0.73	-0.20	0.32	-0.11	-0.72
L461	-0.01	0.15	-0.17	0	-0.03
L462	-0.06	0.13	-0.13	-5E-07	-0.07
E464	0.00	-6.47	6.46	0	-0.01
I465	-0.01	0.17	-0.21	0	-0.05

Table S7: Per residue decomposition of the binding energy for 'OCA' and co-activator in System D. The table contains the decomposition of calculated ΔG_{bind} (kcal/mol) on per residue basis into Vander Waals, electrostatic, polar, and non-polar solvation energy component.

System D (Residue Number)	Vander Waals	Electrostatic	Polar Solvation	Non-Polar Solv.	TOTAL
	ΔE_{vdw}	ΔE_{ele}	$\Delta G_{sol\ GB}$	$\Delta G_{sol\text{-}np}$	ΔH_{GB}
M262	-0.81	-0.65	0.31	-0.15	-1.30
P263	-0.04	-1.50	1.44	0.00	-0.11
Q264	-0.02	-0.79	0.80	0	0.00
T267	-0.18	-1.22	1.28	-0.02	-0.15
L284	-1.41	-0.85	1.18	-0.13	-1.20
M287	-1.85	-0.07	0.61	-0.19	-1.51
A288	-1.09	-0.08	-0.12	-0.10	-1.39
H291	-1.47	0.04	0.50	-0.15	-1.08
V292	-0.35	1.23	-1.15	0.00	-0.28
Q293	-0.69	2.07	-1.06	-0.14	0.18
V296	-1.85	1.74	-1.54	-0.27	-1.92
K300	-1.60	-63.69	61.70	-0.61	-4.20
F305	-0.43	-0.66	0.67	-0.04	-0.45
H310	-2.56	-4.85	6.82	-0.44	-1.03
E311	0.50	-37.09	34.25	-0.24	-2.57
I314	-4.15	1.08	-0.73	-0.62	-4.41
L317	-1.79	-0.19	0.72	-0.23	-1.48

K318	-1.19	4.59	-3.96	-0.20	-0.76
M325	-2.16	-1.94	2.48	-0.24	-1.86
R328	-1.38	9.80	-9.00	-0.15	-0.72
S329	-1.20	-3.31	2.58	-0.13	-2.06
I332	-0.85	1.45	-1.60	-0.15	-1.15
I345	-0.97	0.48	-0.43	-0.10	-1.01
I349	-2.00	0.31	-0.34	-0.20	-2.23
I354	-1.03	0.24	-0.29	-0.11	-1.19
Y358	-1.00	-1.20	0.77	-0.09	-1.51
I359	-0.66	-0.03	0.10	-0.03	-0.61
M362	-1.11	0.17	0.18	-0.05	-0.82
F363	-0.66	0.14	0.35	-0.03	-0.19
Y366	-0.86	-1.51	1.00	-0.10	-1.48
H444	-0.57	-1.92	1.83	-0.04	-0.69
M447	-0.68	0.25	-0.30	-0.04	-0.78
L461	-2.02	-0.49	0.66	-0.35	-2.20
L462	-0.20	0.05	-0.07	0.00	-0.21
E464	-3.03	-27.48	30.68	-0.57	-0.41

Table S8: Per residue decomposition of the binding energy for co-activator in absence of 'OCA' (System C). The table contains the decomposition of calculated ΔG_{bind} (kcal/mol) on per residue basis into VdW, electrostatic, polar and non-polar solvation energy component.

System C Residues number	van der Waals	Electrostatic	Polar Solvation	Non-Polar Solv.	TOTAL
	ΔE_{vdw}	ΔE_{ele}	$\Delta G_{sol\ GB}$	ΔG_{solnp}	ΔH_{GB}
M262	-0.01	0.29	-0.25	0	0.03
P263	-0.01	-0.29	0.28	0	-0.01
Q264	0.00	-0.34	0.35	0	0.007
T267	0.00	-0.26	0.26	0	0.002
L284	-0.01	0.15	-0.14	0	0.003
M287	-0.02	0.38	-0.39	0	-0.023
A288	-0.05	0.28	-0.24	0	-0.01
H291	-0.07	0.62	-0.57	0	-0.02
V292	-1.57	1.33	-0.87	-0.19	-1.30
Q293	-1.38	0.49	0.54	-0.26	-0.61
V296	-2.93	1.44	-1.09	-0.37	-2.94

K300	-1.38	-79.79	76.06	-0.63	-5.73
F305	-0.25	-0.58	0.57	-0.04	-0.30
H310	-0.66	-0.83	1.68	-0.11	0.07
E311	0.67	-21.06	17.76	-0.21	-2.84
I314	-4.06	0.84	-0.49	-0.67	-4.38
L317	-1.74	-0.21	0.90	-0.18	-1.23
K318	-1.60	1.08	-0.33	-0.19	-1.03
M325	-0.02	0.21	-0.17	0	0.01
R328	-0.01	-10.06	10.20	0	0.12
S329	0.00	0.04	-0.02	0	0.01
I332	0.00	0.01	0.00	0	0.00
I345	0.00	-0.15	0.16	0	0.00
I349	0.00	-0.13	0.13	0	0.00
I354	0.00	0.05	-0.05	0	0.00
Y358	0.00	0.06	-0.06	0	0.00
I359	0.00	0.09	-0.09	0	0.00
M362	0.00	0.13	-0.13	0	0.00
F363	0.00	0.09	-0.09	0	-0.00
Y366	0.00	0.12	-0.13	0	-0.00
H444	-0.01	0.18	-0.14	0	0.031
M447	0.00	-0.05	0.06	0	0.007
L461	-1.76	-1.64	2.40	-0.35	-1.34
L462	-0.57	-0.48	0.51	-0.03	-0.57
E464	-1.67	-39.73	41.11	-0.47	-0.76
I465	-2.43	0.22	-0.24	-0.32	-2.77

2. Supplementary Figures

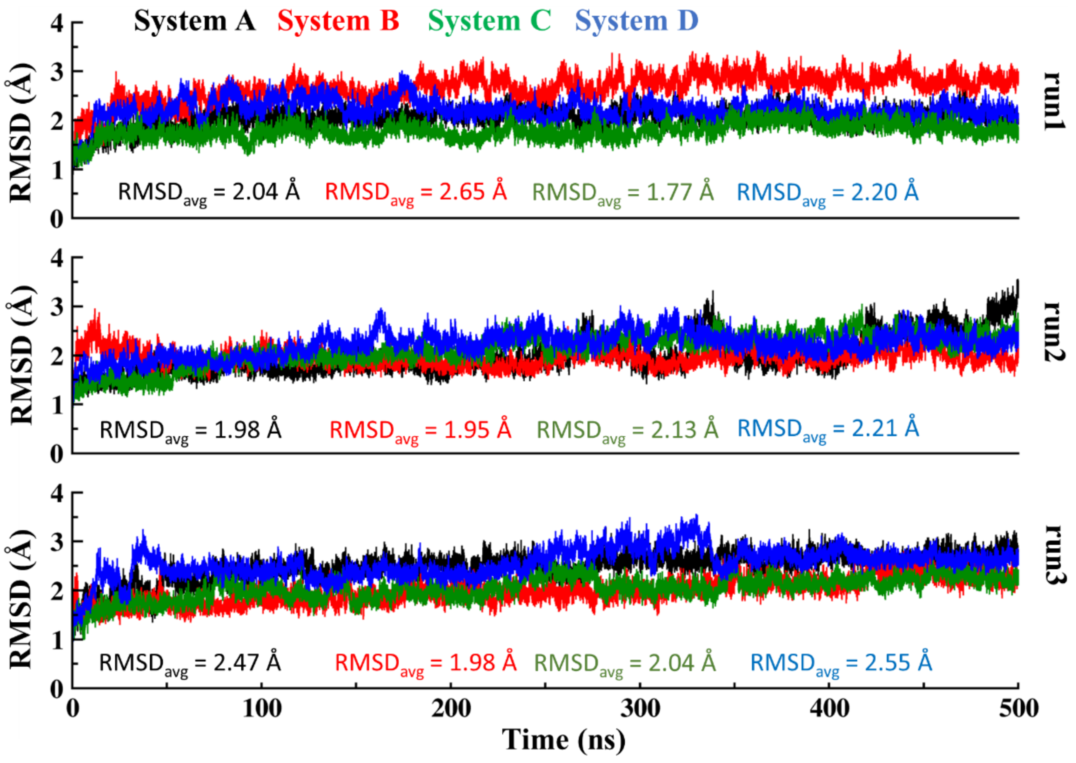

Figure S1: *RMSD plots of triplicate 500ns simulations of FXR systems. Data from the backbone atoms of the three independent runs from different seed values are plotted in black (System A), red (System B), green (System C), and blue (System D) lines. The average RMSD values for each system are shown.*

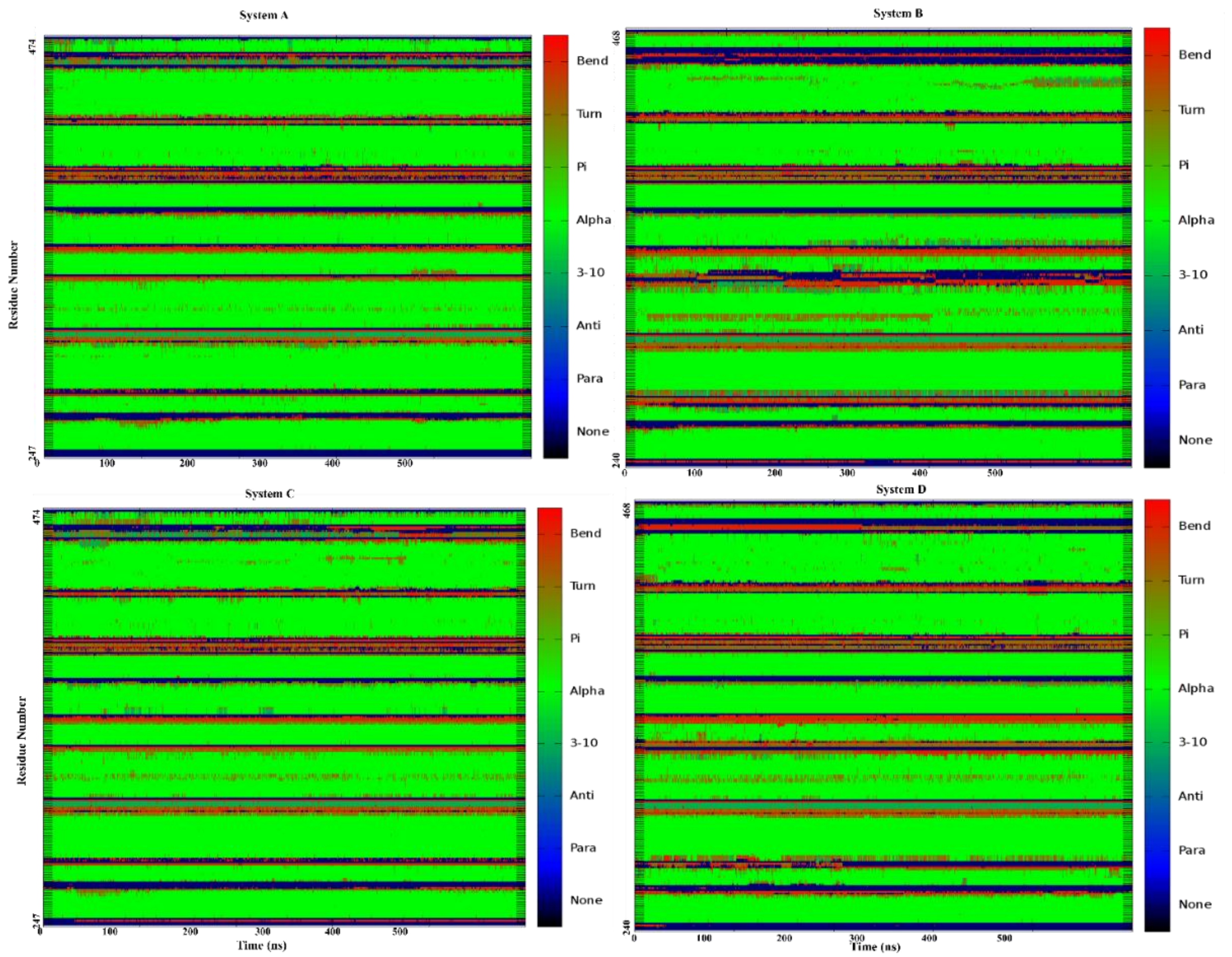

Figure S2: *The DSSP plots for the simulated FXR systems (Systems A-D).*

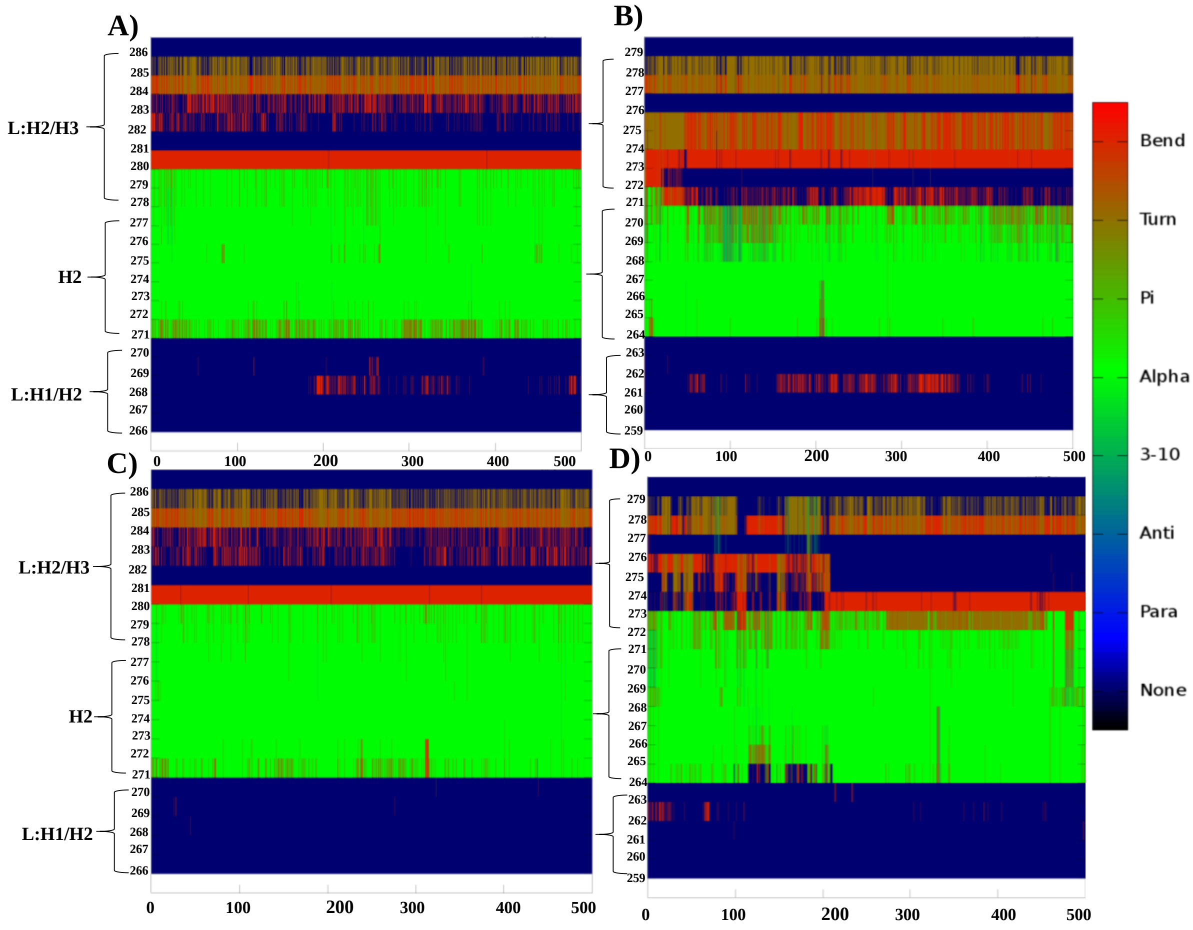

Figure S3: *The DSSP plots for secondary structure transitions in region (helix H2 and loops L:H1/H2 and L:H2/H3) of FXR during 500ns MD simulations in System A, B, C and D, respectively.*

B)

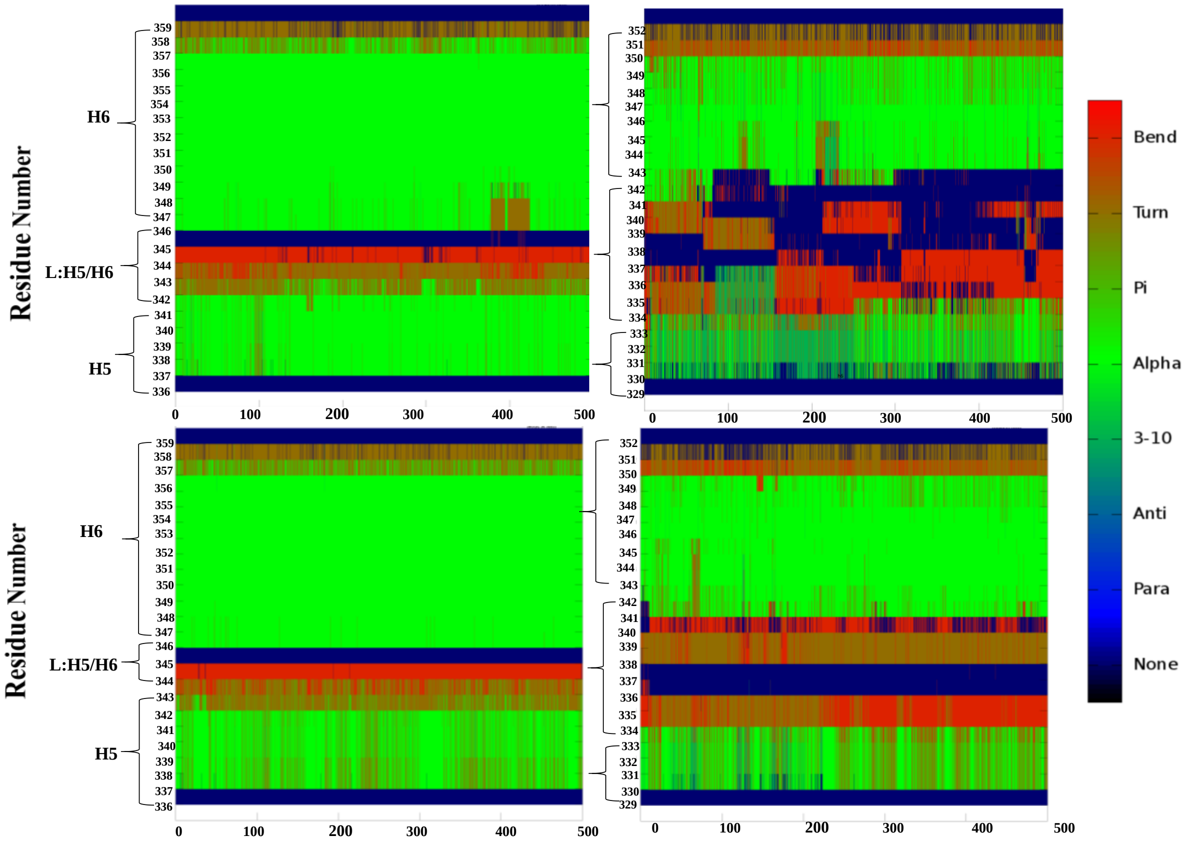

***Figure S4**: The DSSP plots for secondary structure transitions in region (helices H5, H6 and loop L:H5/H6) of FXR during MD simulations in Systems A, B, C and D, respectively.*

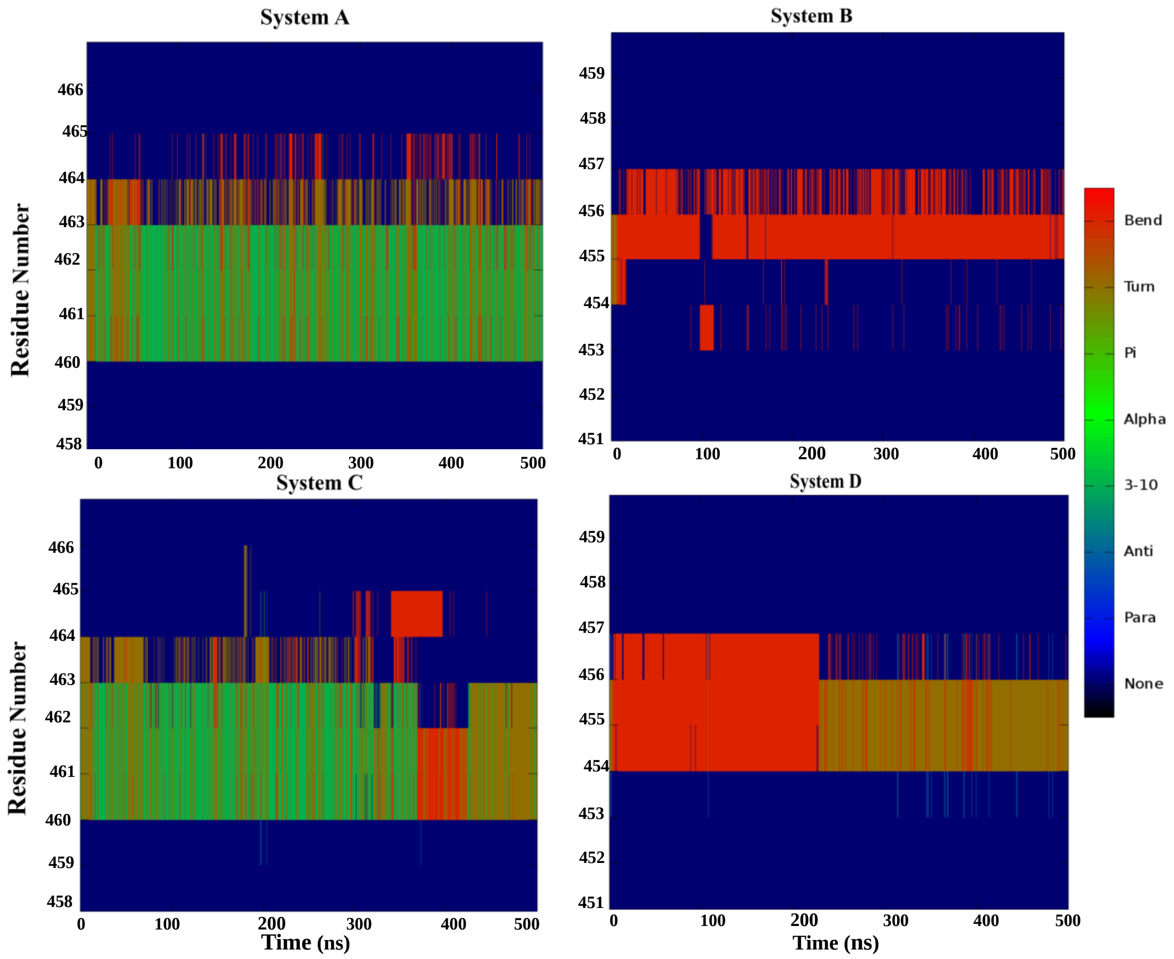

Figure S5: *The DSSP plots for secondary structure transitions in region (loop L:H11/H12) of FXR during MD simulations in Systems A, B, C and D, respectively.*

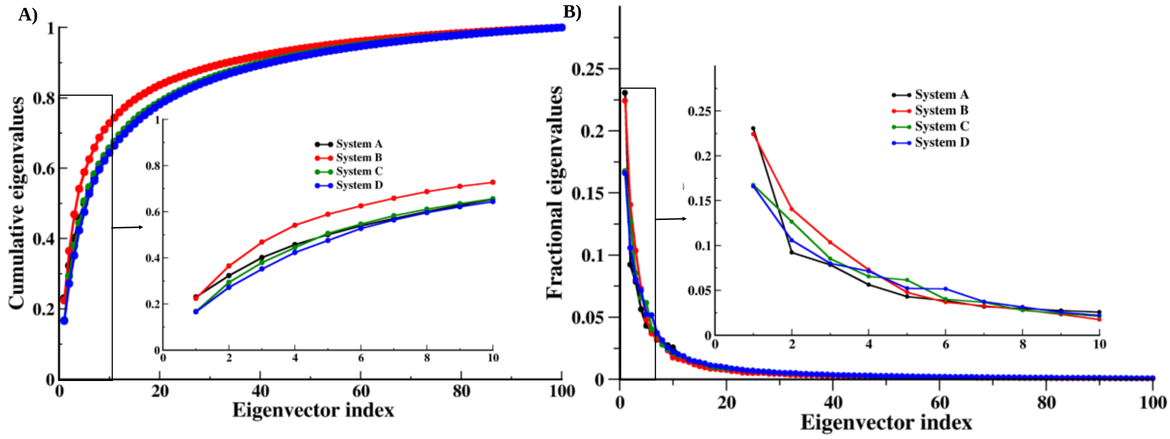

Figure S6: *The overall motion of FXR systems from stable MD trajectories for each system of FXR.* ***A****) Cumulative contribution and eigenvalues of the top 10 PCs in all systems were mentioned. The PC1 represents the largest fluctuation, PC2 is second and so on.* ***B****) The plot*

for fractional eigenvalues for all systems of FXR. The first top PCs were shown with arrow for all systems.

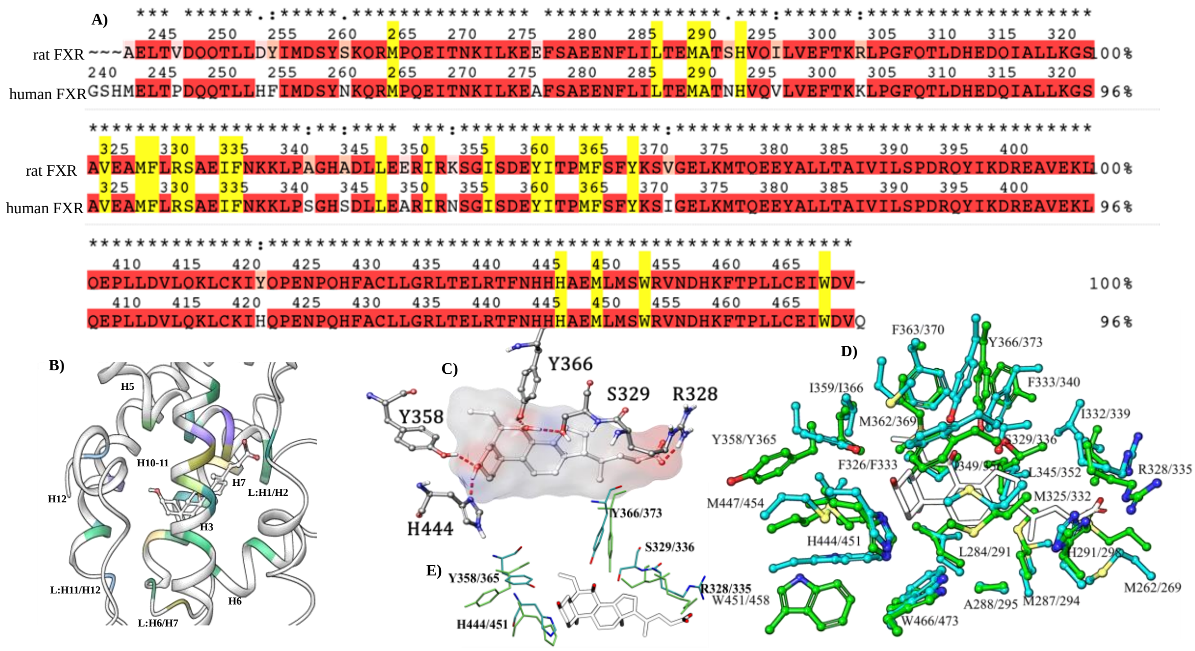

***Figure S7: A)** The interactive residues of FXR involved in binding of 'OCA'. Multiple sequence alignment for the System C (PDB ID: 5Q0K (human)) and System D (PDB ID: 1OSV (rat)). The sequence of mouse shares 96% similarity with the human protein. The binding site residue (within 4.0 Å) are conserved in both rat and human, highlighted in yellow colour **B)** The residues of binding pockets have shown in residue type colors, and the helices involved in the pocket are H3, H5, H6, H7, H10-11, H12 and loops between the L: H1/H2, L: H6/H7 and L:H11/H12. **C**) The electrostatic view of the 'OCA' and the residue R328, S329, Y358, Y366, and H444 are shown in CPK presentation and the red dotted line depicted the hydrogen bond (HB) interaction. **D**) The superimposition of the interacting residue of FXR in C and D Systems were shown. **E**) The significant change in the conformation of the binding site residues is depicted in both Systems C (lime) and D (cyan).*

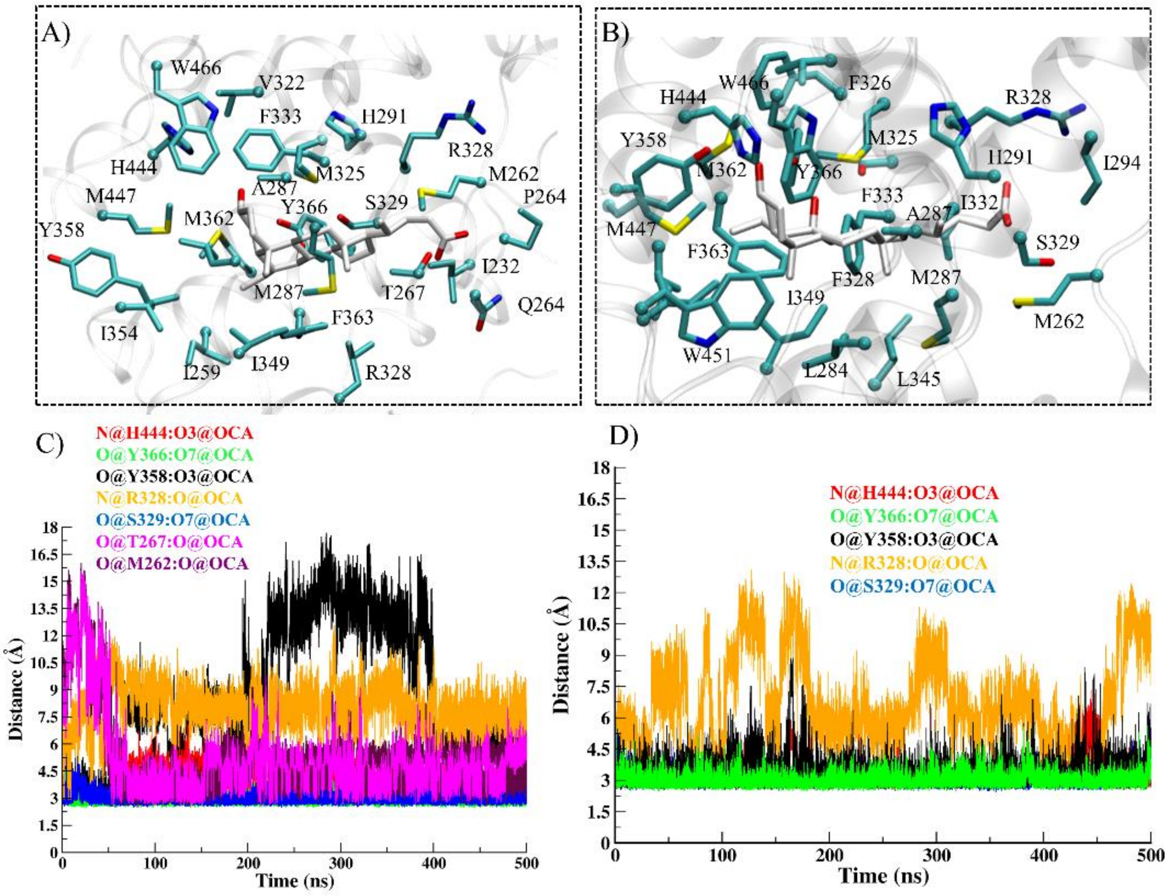

Figure S8: *The interacting residue at the binding site of the 'OCA' in FXR.* ***(A*** *and* ***B****) The interacting residues within 4.5Å of binding pockets of the FXR stable MD state of Systems B and D were shown.* ***(C*** *and* ***D)*** *The stability analysis of HB pairs N@R328: O@OCA, O@S329:O7@OCA, O@T267:O@OCA, O@Y358:O3@OCA, O@Y366:O7@OCA, N@H444:O3@OCA, and O@M262: O@OCA in both Systems B and D during MD simulation.*

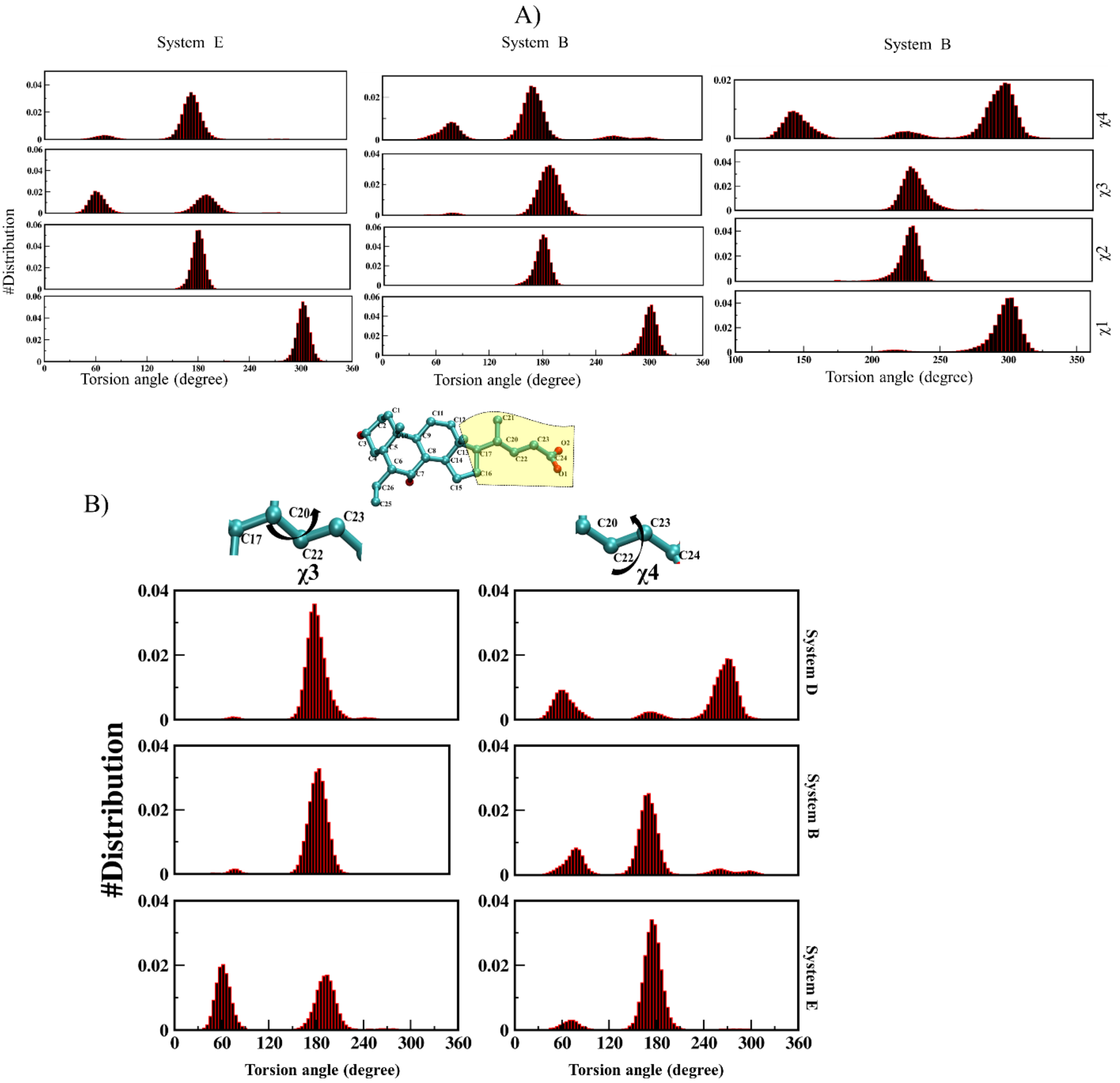

Figure S9: Changes in the chi angle of 'OCA' with respect to time during the simulation. A) *The key ''OCA'' dihedral angles are highlighted and studied throughout the simulation* ***B)*** *The frequency distribution of torsion angle (3 and 4) during MD simulation in solvent System (System E), System B and System D are shown. Bar plots summarize the conformation of the torsion throughout MD simulation.*

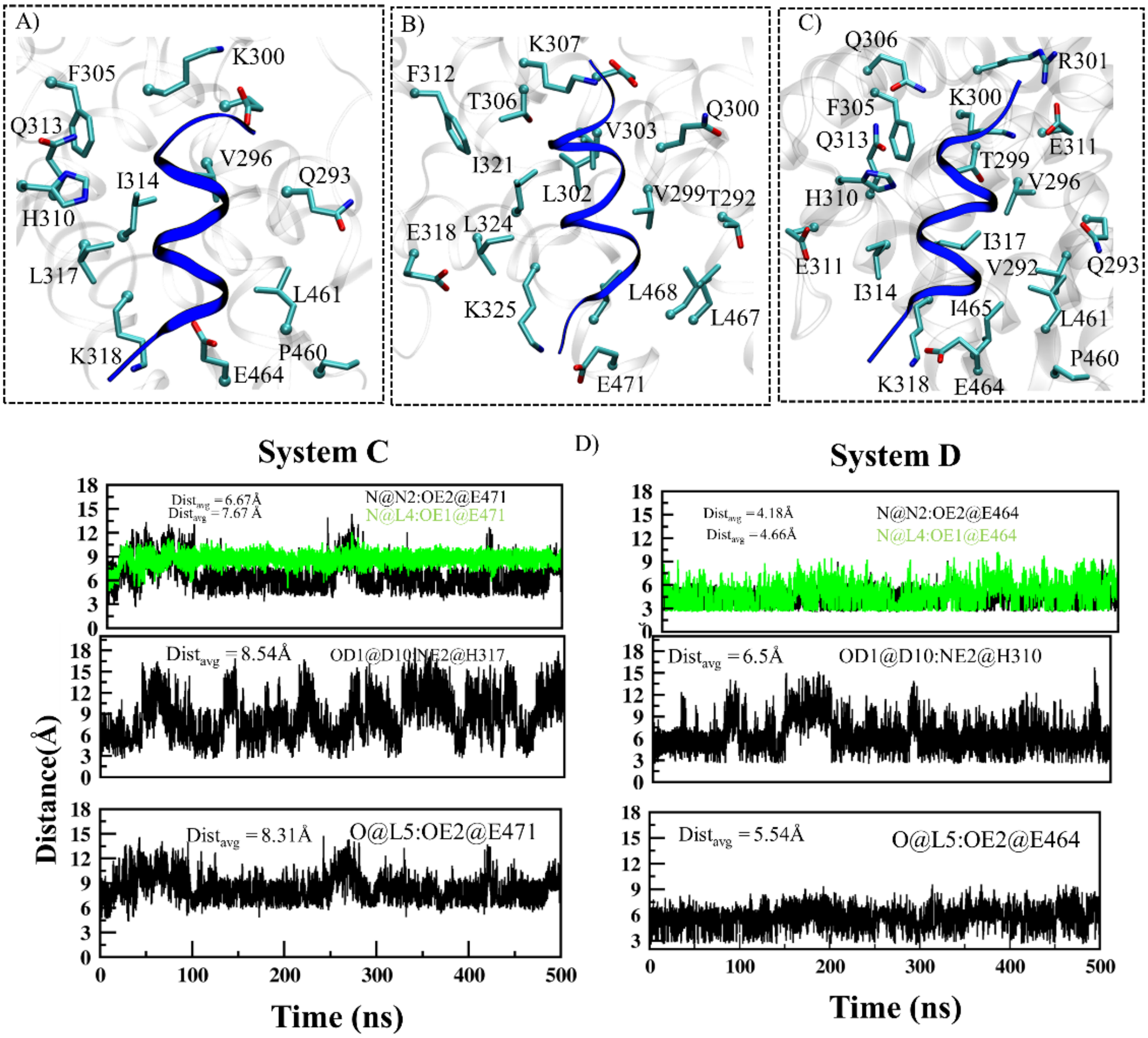

Figure S10: ***A****) The interacting residue at the binding site of the 'OCA' in FXR.* ***(B, C****) The interacting residues within 4.5Å of 'OCA' in the stable MD states of Systems B and D, respectively, were shown.* ***D****) The panel depicts the HB distances in Systems C and D, throughout MD simulation.*

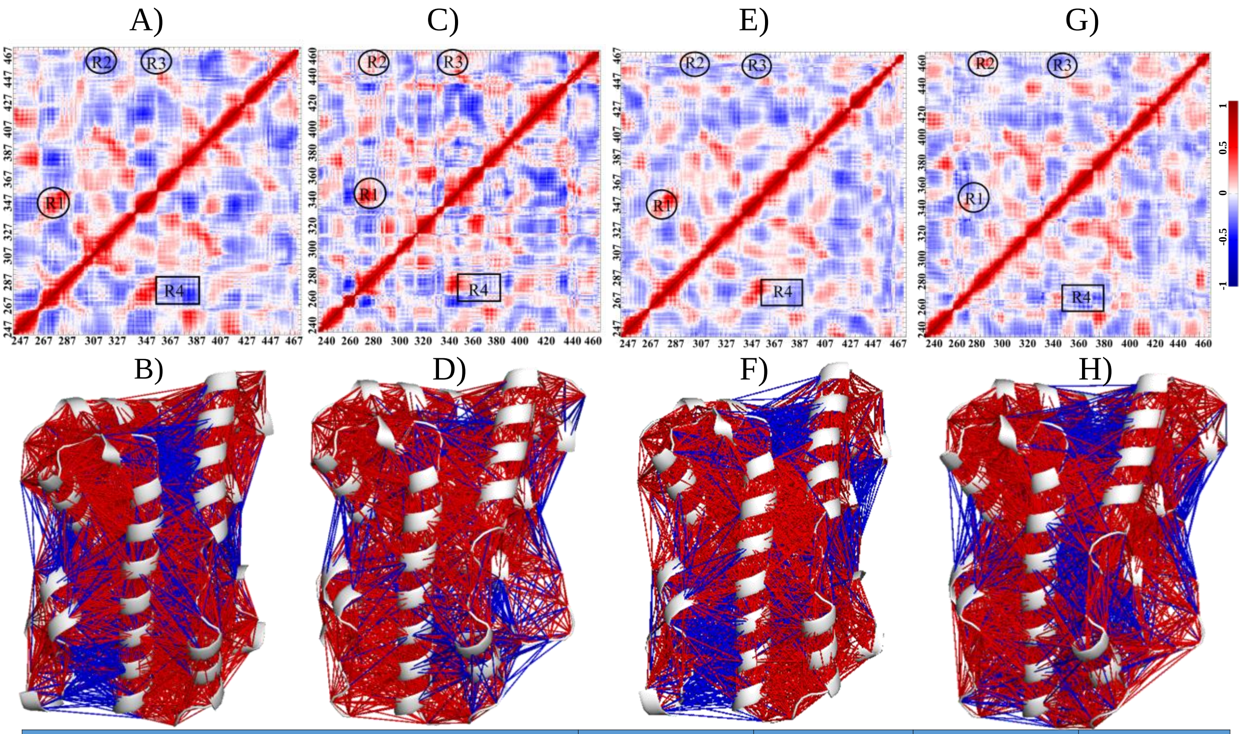

I)

Regions	System A	System B	System C	System D
**R1 (H2, L:H2/H3, H3 and H6, L:H6/H7)**	+	+	+	-
**R2 (H3, L:H11/H12 and H12)**	-	+	-	+
**R3 (H6, L:H6/H7, H7 and L:H11/H12, H12)**	-	+	-	-
**R4 (H6, L:H7/H8, H7, H8 and L:H1/H2, H2,L:H2/H3, H3)**	-	+/-	-	-

Figure S11*: Intra-protein dynamic motion of FXR in different simulated systems. The 2D and 3D representation of the Cα fluctuations around their mean position during MD simulation for (****A****,* ***B****) System A, (****C****,* ***D****) System B, (****D****,* ***F****) system C and (****G****,* ***H****) System D, where red and blue line indicates the corelated and anti-corelated motions, respectively. The regions labelled are R1 (267-287 and 345-363:H2,H3,L:H2/3 and H6,L:H6/H7 and H7), R2 (287-307 and 450-463:H3 and L:H11/H12), R3 (347-367 and 450-468: H6,L:H6/H7 and L:H11/H12 and H12) and R4 (344-386 and 262-280:H6 ,H7,H8, L:H6/H7 and L:H1/H2, L:H2/H3,H2 and H3) (****I****) In the table the '+' and '-' signs, depicting the correlated and anti-correlated motions, respectively.*

3. Supplementary Results

3.1 Torsion angle analysis

To substantiate this, we performed the torsion angle analysis for the flexible region of 'OCA' (tail region) in Systems B, D, and E. Bar plots summarized the conformation of the torsion throughout the course of MD simulation. We mainly considered the dominant torsions in angle chi3 and chi4 for three FXR systems (**Figure S9B**). It is observed that torsion angle chi3 shows more torsions between the 60° to 180° in System E as compared to B and D. The angle chi4 has torsions between the 60° to 300° in Systems B as compared to Systems D and E.

3.2 Internal dynamics of FXR conformational diversity

The internal motion of the FXR LBD is explored by using DCCMs plots in all four systems (**Figure S**) .in which the positive regions (marked in red), indicate the strongly correlated motions of residues (C_{ij} =1), whereas the negative regions (marked in blue) are associated with the anticorrelated movements (C_{ij}= -1). The regions with significant variations are highlighted in (**Figure S**). From the DCCM analysis, it can be seen that the presence of 'OCA' and 'co-activator' both produced strongly correlated and anti-correlated motions in FXR (System D) than the other systems. In Systems A to C, we observe that in region R1, residues of helices H2, H3 and loop L: H2/H3 move with correlated motion with the residues of helix H6 and loop L: H6/H7 as compared to System D. The residues of the region R2 of H3 shows the anti-correlated motion with the residue of loop L: H11/H12, helix H12 in Systems A and C and correlated motion in Systems B and D. The region R3 of helix H6 and loop L:H6/H7 shows correlated motion with the residues of loop L:H11/H12 and helix H12 in System B as compared to other three systems. The regions R4 of helices H6, H7, H8, and loop L:H6/H7 display anti-correlated motion in Systems A, C and D with the loops L:H1/H2, L:H2/H3, and helices H2, H3 and mixed motion in System B. The interesting region is R2 and R3 as it has loop L: H11/H12 and helix H12, in which it is noticeable that the presence of 'OCA' has increased the coordinated and correlated motion in the overall FXR structure as compared to the other systems.
